# A collection of yeast cellular electron cryotomography data

**DOI:** 10.1093/gigascience/giz077

**Published:** 2019-06-27

**Authors:** Lu Gan, Cai Tong Ng, Chen Chen, Shujun Cai

**Affiliations:** Department of Biological Sciences and Centre for BioImaging Sciences, National University of Singapore, 14 Science Drive 4, Singapore 117543; Department of Biological Sciences and Centre for BioImaging Sciences, National University of Singapore, 14 Science Drive 4, Singapore 117543; Department of Biological Sciences and Centre for BioImaging Sciences, National University of Singapore, 14 Science Drive 4, Singapore 117543; Department of Biological Sciences and Centre for BioImaging Sciences, National University of Singapore, 14 Science Drive 4, Singapore 117543

**Keywords:** yeast, chromatin, nucleus, cryo-ET, cryo-EM, template matching, mining

## Abstract

**Background:**

Cells are powered by a large set of macromolecular complexes, which work together in a crowded environment. The *in situ* mechanisms of these complexes are unclear because their 3D distribution, organization, and interactions are largely unknown. Electron cryotomography (cryo-ET) can address these knowledge gaps because it produces cryotomograms—3D images that reveal biological structure at ~4-nm resolution. Cryo-ET uses no fixation, dehydration, staining, or plastic embedment, so cellular features are visualized in a life-like, frozen-hydrated state. To study chromatin and mitotic machinery *in situ*, we subjected yeast cells to genetic and chemical perturbations, cryosectioned them, and then imaged the cells by cryo-ET.

**Findings:**

Here we share >1,000 cryo-ET raw datasets of cryosectioned budding yeast *Saccharomyces cerevisiae*collected as part of previously published studies. These data will be valuable to cell biologists who are interested in the nanoscale organization of yeasts and of eukaryotic cells in general. All the unpublished tilt series and a subset of corresponding cryotomograms have been deposited in the EMPIAR resource for the community to use freely. To improve tilt series discoverability, we have uploaded metadata and preliminary notes to publicly accessible Google Sheets, EMPIAR, and GigaDB.

**Conclusions:**

Cellular cryo-ET data can be mined to obtain new cell-biological, structural, and 3D statistical insights *in situ*. These data contain structures not visible in traditional electron-microscopy data. Template matching and subtomogram averaging of known macromolecular complexes can reveal their 3D distributions and low-resolution structures. Furthermore, these data can serve as testbeds for high-throughput image-analysis pipelines, as training sets for feature-recognition software, for feasibility analysis when planning new structural-cell-biology projects, and as practice data for students.

## Background

Electron cryotomography (cryo-ET) is the combination of electron cryomicroscopy (cryo-EM) and computed tomography. In a cryo-ET experiment, 2D cryo-EM data are incrementally recorded while the sample is rotated by typical angular steps of 1° to 3° over a range of −60° to +60°. These “tilt-series” images are then mutually aligned and combined to generate a 3D reconstruction called a cryotomogram. Because the cryotomogram contains a single field of view, cryo-ET is particularly valuable for the structural analysis of unique objects that cannot be averaged, such as cells [1–3]. A cryotomogram can contain a piece of tissue, a whole cell, a portion of a cell, an isolated organelle, or a field of purified macromolecular complexes. This Data Note focuses on cryo-ET data of cryosectioned cells.

Cryo-EM is becoming a “big data” method [4]. Highly automated cryo transmission electron microscopes, automated data-collection software, and fast-readout direct-detection cameras can now generate terabytes of data per day [5–11]. Cryo-EM “single-particle analysis” (SPA) raw data contain many copies of conformationally and constitutionally similar macromolecular complexes that are suspended in buffer. In contrast, cellular cryo-ET raw data contain many different species of macromolecular complexes. Furthermore, cellular cryo-ET data are usually recorded at lower magnification than for SPA. This dichotomy reflects (with exceptions) different goals: SPA studies aim to achieve maximum resolution of a few species of macromolecular complexes while cellular cryo-ET studies aim to determine how macromolecular complexes are distributed and organized in their intracellular environment. SPA and cellular cryo-ET studies do share similarities. Notably, only a small percentage of the collected data contribute to the published models.

Our group has collected hundreds of tilt series per project. Because our studies are focused on 1 or a few types of structures, most of our data are in surplus. Two types of surplus data are “byproducts,” i.e., imaged cell positions that lack the targeted structures, and “bystanders,” i.e., imaged cellular structures nearby to the targeted structures. We have previously shared cryo-ET data with collaborators and colleagues using commercial internet solutions such as Dropbox and Google Drive, but we found that these tools were suboptimal for sharing multi-gigabyte files. Alternative web technologies have allowed resources such as Electron Microscopy Public Image Archive (EMPIAR) [12] and the Caltech Electron Tomography Database (ETDB-Caltech) [13, 14] to share terabyte-sized datasets globally and more conveniently. We have deposited our published and surplus cryo-ET tilt-series data in EMPIAR.

## Context

**Figure 1:**
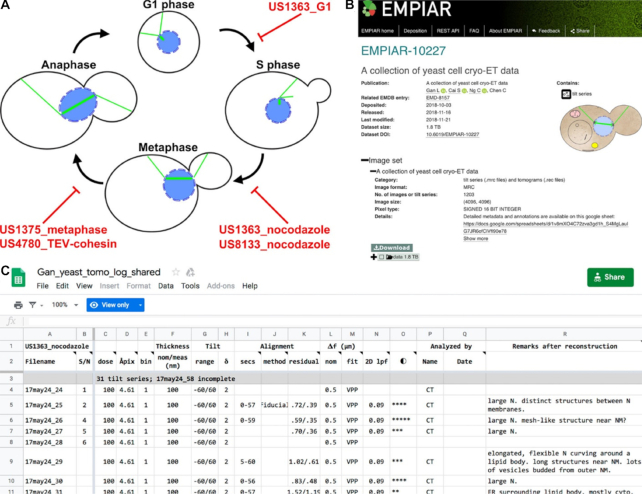
Yeast cryo-ET dataset summary. Yeast cell-cycle stages sampled by these data. The red text indicates the strain ID plus either the cell-cycle state or treatment. **(B)** Screenshot of the EMPIAR entry. Downloads are faster and more reliable when done with the recommended client (Aspera Connect, as of this writing). **(C)** Screenshot of the preliminary notes, which are shared in Google Sheets tabs named after the red text in panel A. The metadata are also available from EMPIAR [20] and GigaDB [21] as a Microsoft Excel spreadsheet file.

We are interested in the relationship between macromolecular structure and function inside cell nuclei. As a model system, we use yeast cells that are arrested at well-defined points in the cell cycle (Fig. 1A). We have shown that chromatin is packed irregularly, without forming any monolithic condensed structures in both interphase and mitosis [15, 16], and that the majority of outer-kinetochore Dam1C/DASH complexes assembles as partial rings and does not contact the kinetochore microtubules' curved tips *in situ* [17]. These studies show that the intracellular distribution and organization of macromolecular complexes are not always consistent with the models derived from *in vitro* studies. Indeed, our efforts to locate Dam1C/DASH *in situ* were hampered because we originally searched for complete rings resting against curved microtubule protofilaments. We also had difficulty locating condensed chromosomes in fission yeast because we were expecting to find a monolithic nucleosome mass separated from a relatively “empty” nucleoplasm.

Our group has recorded >1,000 tilt series of cryosectioned yeast cells. These include the budding yeast *Saccharomyces cerevisiae* and the fission yeast *Schizosaccharomyces pombe*. Only a minority of our tilt series were presented in a paper; this data subset is already available at EMPIAR. Here we present the surplus cellular tilt series data we collected as part of those published studies. We have neither analyzed nor intend to analyze in detail the vast majority of these data. These data will be valuable to other groups interested in macromolecular complexes and cytological features both within and outside the nucleus. Because the typical cryotomogram has ~4-nm resolution, many structures can be identified on the basis of their shape, size, and intracellular context. The cryotomographic densities of some of these structures may contain features that are difficult to see in EM images of plastic sections. Notable examples are nucleosomes and some of the smaller or thinner components of the chromosome-segregation and cell-division machineries.

## Dataset Format and Logistics

All cryo-ET data files are saved in the MRC format [18] under the accession code EMPIAR-10227 (Fig. 1B). Each dataset has a unique name that combines the date of data collection and a serial number. For example, 18jun04a__02 is the second tilt series collected on 2018 June 4, session “a.” Future depositions may use the alternative YYYYMMDD_SN format, making the previous example 20180604_02. Some datasets include both a tilt series and a cryotomogram. The filename extensions follow IMOD conventions: “.mrc” for tilt series and “.rec” for cryotomograms. To conserve storage space and speed up file transfers, the tilt series and cryotomograms have been compressed with lbzip2 and therefore have the “.bz2” extension. The current entry does not include any movie or electron-counted data. In the future, electron-counted raw data will be stored as LZW-compressed .tiff files. If possible, we will add the newer data to the existing entry EMPIAR-10227. Otherwise, we will create new EMPIAR entries that each contain a link to this article.

The pixel sizes used in the present data range from 4.6 to 9.1 Å. Most of the data were recorded on direct-detection cameras that have ~16 million pixels in a 4,096 × 4,096 pixel array. The typical field of view therefore ranges from ~2 to 4 μm^2^. Most tilt series consist of ~61 images because we typically use a ±60° tilt range and a 2° tilt increment. The pixel intensity values in most tilt-series data are stored as 16-bit unsigned integers, so the typical uncompressed tilt series is ~2 gigabytes.

We have shared via Google Sheets [19], EMPIAR [20], and GigaDB [21] a set of tabbed spreadsheets that contain metadata and preliminary notes and observations (Fig. 1C, also see Availability section). The Google Sheets are “live” documents and will be updated as new datasets are deposited and as errata are brought to our attention and then corrected. The first spreadsheet tab has a summary of all the data, links to additional related resources, commonly used commands, and a link to an online feedback form. Subsequent spreadsheet tabs contain detailed information on each tilt series, grouped by a strain ID and a treatment condition. For example, the “US1363_nocodazole” spreadsheet describes cryo-ET data of US1363 cells that were treated with the tubulin-polymerization inhibitor nocodazole.

In the detailed metadata spreadsheets, each row corresponds to 1 tilt series. Some cells were imaged by serial cryo-ET and therefore have the sequence number of each contributing tilt series noted in the “S/N” column. The other columns organize the data-collection parameters, appraisal of image contrast, diagnostic remarks on the data-collection session and quality, and a guess about the cytological features and macromolecular complexes present in the imaged cell. During the initial annotation, we used abbreviations to denote organelles and other cellular features. These abbreviations are defined in the spreadsheet “notes,” which can be displayed by hovering the mouse cursor over the spreadsheet title cell. The accuracy of some of our annotations of cytological features is limited by our current cryo-ET and cell-biology knowledge but will improve with both experience and especially user feedback. We anticipate that cell biologists will use the sorting function to shortlist the tilt series most salient to their studies.

## Methods

Cells were either grown in conditions that arrest populations at defined stages of the cell cycle or treated with drugs to perturb their cytology and cell-cycle progress. Because of our interest in mitosis and chromosome condensation, the present data capture cells in G1 phase, metaphase, and in mitosis with disrupted mitotic spindles. Liquid-cultured cells were collected by either centrifugation or vacuum filtration. These cells were then either high-pressure frozen or self-pressurized frozen in the presence of the extracellular cryoprotectant dextran. The frozen-hydrated cell block was sectioned in a cryomicrotome, producing a ribbon of cryosections. This cryosection ribbon was attached to either a continuous- or holey-carbon EM grid, which had been pre-coated with 10-nm-diameter gold nanoparticles. Nanoparticles serve as easy-to-locate fiducial markers that facilitate tilt-series alignment. The cell cryosections were then imaged on a Titan Krios equipped with a direct detector, with or without Volta phase contrast. Additional details can be found in our earlier papers [15–17] and in the online spreadsheets.

Cryotomogram reconstruction, visualization, and analysis were done on a modern workstation computer with popular open-source software (Table 1). Radiation damage causes some cryosection positions to undergo non-uniform distortions, meaning that alignments were done using only the fiducials proximal to the structure of interest. Most of the tilt series were aligned using 4−12 fiducials coincident with the nuclei. To improve the visualization of other features, users should do a “local” alignment using only the fiducial markers closer to their structures of interest. If local alignment is not desired, the tilt series can be semi-automatically aligned using fiducials spread throughout the field of view and then reconstructed using software like Etomo and Protomo [25, 26]. Such cryotomograms tend to have uniform resolution at all positions where the cryosection is in contact with the carbon substrate.

**Table 1:** Recommended hardware and software

Tool	Recommendation	Notes
Computer	Modern workstation	More memory (RAM) facilitates comparisons of multiple cryotomograms.
Display	≥27 inch monitor	
Operating system	Linux	Most cryo-EM software is developed on Linux; extra effort is needed to run this software in macOS or Windows.
Visualization software	3dmod (IMOD)	FIJI can also be used, but it is not optimized for tomography data.
Reconstruction software	Etomo (IMOD)	A solid-state disk and a CUDA-compatible NVIDIA GPU are highly recommended.
Download client	AsperaConnect	This software enables fast, fault-tolerant downloads from EMPIAR.
Notes	Google Sheets, Microsoft Excel	The shared spreadsheet can be downloaded and then customized.

FIJI: Fiji Is Just ImageJ.

Cryotomograms are noisier than SPA reconstructions, meaning that these datasets are very difficult to comprehend when visualized as isosurfaces. Instead, cryotomograms should be visualized as cryotomographic slices: 2D images that average multiple voxels along 1 axis. The slice thickness should match the structure of interest, e.g., 10 nm for nucleosomes. To facilitate comparison between datasets, multiple cryotomograms can be loaded into random-access memory (RAM) in 1 instance of the program 3dmod [25]. Assuming they all “fit” into memory, cryotomograms loaded this way can be rapidly toggled in sequence using the “1” and “2” shortcut keys.

Reconstructed cryotomograms are usually the starting point of more quantitative analysis. Examples of deeper analysis by template matching, classification, and subtomogram averaging can be found in recent reviews and the many excellent papers cited within [28–31]. Because structural cell biology is a new field, most of our studies have required new analysis tools. We have written a number of python scripts to facilitate the 3D packing analysis of subtomograms [32]. These scripts control programs from published image-analysis packages [25, 33–35], most of which are open source.

## Data Validation and Quality Control

The yeast cryo-ET data have been recorded under a variety of conditions (magnification, dose, tilt increment, defocus) with different contrast mechanisms (defocus phase contrast vs Volta phase contrast). Furthermore, the tilt series have differences in quality due to variations in freezing, attachment to the grid, radiation damage, or a combination of these factors. Owing to this variability, we cannot assign a single validation metric to the entire set of tilt series. We have qualitatively assessed each tilt series' contrast relative to others recorded in the same session (tens of tilt series per session). The contrast is rated from 1 to 5 stars and is recorded in the online spreadsheet columns marked with the “
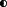
” symbol. The 4- to 5-star data typically reveal features such as membrane leaflets, clear separation of nucleosome-like particles, and particles smaller than nucleosomes. These evaluations were made from cryotomograms when possible.

The deposited cryotomograms should be considered preliminary for 3 reasons. First, most of the cryotomograms were reconstructed using the subset of fiducial markers coincident with the nucleus, which results in lower reconstruction quality elsewhere in the cell. Second, the fiducial centers were manually fine-tuned for the few tilt series that contributed to the final published figures. Third, we anticipate that future developments in fiducial-assisted and fiducial-less alignment will produce better cryotomograms than currently possible.

## Re-use Potential

**Figure 2:**
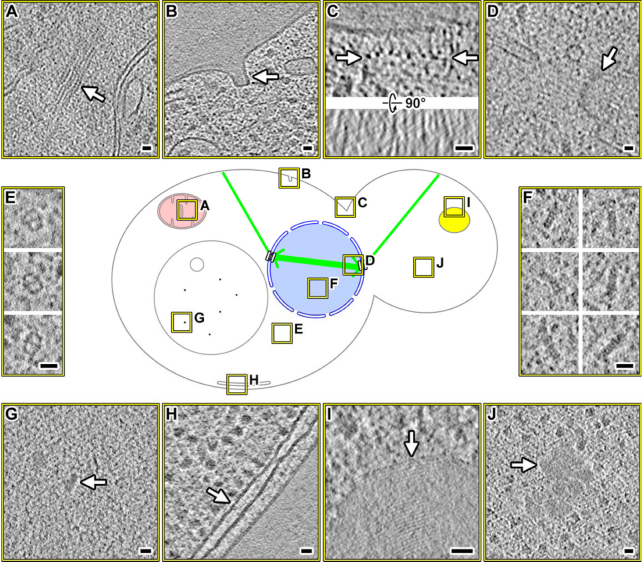
Easy-to-find structures in yeast cryotomograms. Center: graphical legend showing the locations of interesting features (boxed in yellow), which are enlarged as cryotomographic slices (10–20 nm thick). **(A)** Filament bundle within a mitochondrion. **(B)** Eisosome; see [22] for identification details. **(C)** Cytokinetic machinery. Upper panel: transverse view. The row of filamentous complexes is indicated by arrows. Lower panel: longitudinal view of the filaments. See [23] for examples of fission-yeast cytokinetic machinery. **(D)** Microtubule-organizing center. **(E)** Fatty-acid synthases. **(F)** Intranuclear proteasomes; see [ 24] for examples of algal intranuclear proteasomes. **(G)** Particles inside a vacuole. **(H)** Endoplasmic reticulum adjacent to the plasma membrane. **(I)** Lipid-droplet-like body with periodic internal structure. **(J)** Amorphous cytoplasmic aggregate. Scale bar = 20 nm in all panels.

The deposited yeast cryo-ET data contain a large number of easy-to-find or abundant organelles and macromolecular complexes such as mitochondria, eisosomes, cytokinetic machinery, microtubule-organizing centers, fatty-acid synthases, proteasomes, vacuoles, rough endoplasmic reticulum, lipid bodies, and cytoplasmic amorphous aggregates (Fig. 2). Closer inspection may reveal poorly documented subcellular features. Examples of such features include mitochondrial internal filaments (Fig. 2A) and ordered layers in lipid-droplet-like bodies (Fig. 2I). Cell biologists may want use these data to measure local concentrations of macromolecular complexes, detect interactions between these complexes, determine the orientations of large complexes *in situ*, test for the existence of putative cellular features, and determine how cellular bodies make direct contact with one another. Furthermore, these data will provide morphological, distance, or stoichiometric constraints for researchers who are attempting to reconstitute either a complex or a cellular body.

If multiple copies of a macromolecular complex can be detected in ≥1 cellular cryotomograms, they can be computationally extracted and then analyzed as “single particles.” In this subtomogram-averaging approach, the subtomograms are aligned and then averaged together to create density maps that have higher-resolution features visible, as discussed in recent reviews [28–31]. The centers of mass and orientation information can then be used to remap the average back into a volume the same size as the cryotomogram. If the complexes are densely packed, these remapped models will reveal higher-order structure as seen in polysomes and oligonucleosomes [36, 37].

**Figure 3:**
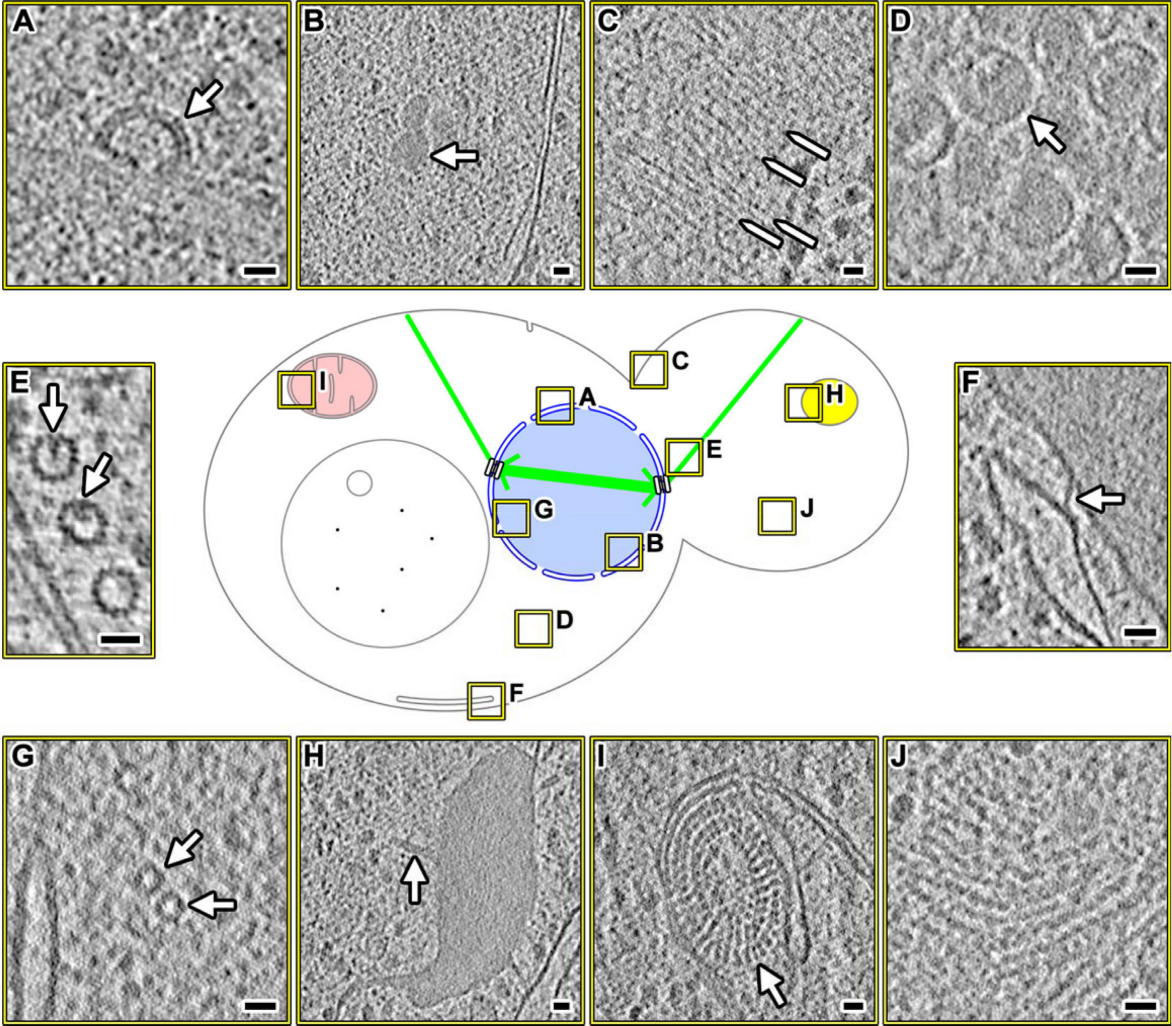
Hard-to-find structures in yeast cryotomograms. Center: graphical legend showing the locations of interesting features (boxed in yellow), which are enlarged as cryotomographic slices (10–20 nm thick). **(A)** A coated pit-like structure, docked to the outer nuclear membrane. **(B)** Intranuclear granule. **(C)** Septin-like cytokinesis machinery. A few examples are indicated by the pointed lines. These filaments run parallel to the mother-daughter cell axis. **(D)** Virus-like particles in the cytoplasm. **(E)** Lumenal particles in cytoplasmic microtubules. **(F)** Connection between the endoplasmic reticulum and plasma membrane; see [27] for more details about these inter-membrane connections. **(G)** Short intranuclear 15-nm-diameter tubes. **(H)** A lipid body with thin protrusions, one of which is indicated by the arrow. **(I)** Mitochondrial periodic structures extending from the inner membrane into the matrix. **(J)** Filamentous cytoplasmic aggregates.

Cellular cryotomograms also contain hard-to-find structures (Fig. 3). These structures are either rare or located in cellular positions that we rarely target, such as the bud neck (Fig. 3C). Many of these structures, such as inter-membrane contact sites (Fig. 3F) and lipid-body protrusions (Fig. 3H), are poorly documented in the cryo-ET literature. We anticipate that yeast cryo-ET data will help stimulate the discovery and detailed characterization of interesting eukaryotic subcellular bodies just as cellular cryo-ET has done for bacterial cell biology [38–42]. Furthermore, structures that are identified by other groups can be retrospectively analyzed in these data in the context of known cell-cycle states and pharmacological perturbations.

Users should note that we arrested the yeast cells in various cell-cycle stages to allow comparative studies of nuclear structures such as chromatin, spindles, and kinetochores. Because the cell cycle affects the entire proteome, this dataset will shed light on how other organelles and cytoplasmic macromolecular complexes are cell-cycle regulated. Some of the structures observed in these yeast data may also be stress induced. Indeed, recent studies showed that upon starvation, eukaryotic translation initiation factor 2B forms large filament bundles in budding yeast [43, 44].

These data span a range of defoci and magnifications, with or without the Volta phase contrast [45]. Such experimental diversity will allow software developers to test the robustness of new image-processing routines used in automated alignment [25, 26], template matching (also called 3D particle picking), subtomogram averaging, and classification [35, 46–49]. The yeast cryo-ET data can also be used to train machine-learning algorithms to detect features in both tilt series and cryotomograms [50–52]. Furthermore, data-sharing resources may use these data to develop annotation and browsing tools [53–55].

The vast majority of our cryo-ET imaging was recorded with first-generation direction-detection cameras, without energy filtering. If either the structure of interest or a structure of equivalent size can be detected in the present data, then it will most certainly be detectable in data recorded on electron-counting cameras, both with or without energy filtering. Therefore, these data will facilitate feasibility analyses.

Finally, new structural cell biologists will find these data useful as real-world examples that complement the lessons from cryo-EM tutorials [56, 57]. The vast majority of the deposited data are from grids that have gold nanoparticles, making the alignment process similar to—and therefore a direct follow-on to—the IMOD plastic-section tutorial dataset [53]. Students can use the reconstructed cryotomograms to practice manual annotation and more automated analyses such as template matching and subtomogram averaging.

## Availability of supporting data and materials

We have deposited our data under accession code EMPIAR-10227 [20]. We excluded “unusable” tilt series, which have ≥1 of the following image or sample properties: extreme drift, occlusion by large ice crystals, cracks in the ice or carbon substrate, or completely detached sections. We also included a copy of the tilt series that were already deposited as part of our original research papers. Key metadata are available in Google Sheets [19] and EMPIAR [20], which can be copied to the user's own Google Drive or downloaded as a Microsoft Excel spreadsheet file. Thereafter, the user can sort the rows to identify smaller subsets of tilt series that have the desired properties or structures. Python scripts to help facilitate 3D packing analysis of subtomograms are available in the ot-tools GitHub repository (RRID:SCR_017191) [ 28]. A copy of the metadata spreadsheets and the ot-tools scripts are available in the *GigaScience* database, GigaDB [21]. We note that anyone can add our data to an ETDB database [14] and thereby enable the numerous benefits of ownerless-ledger metadata and decentralized storage. Feedback can be sent via a Google Form [58].

The tilt series and cryotomograms are organized in the following directory structure:

**Table:** 

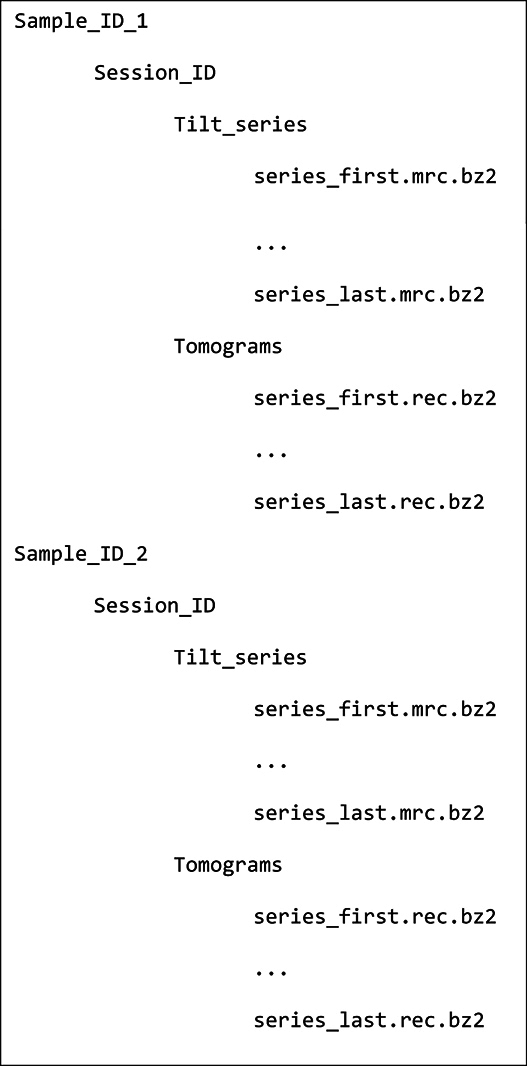

These data can be downloaded either using the Aspera Connect client or with a web browser. We do not recommend download by web browser due to its slow speed and lack of fault tolerance. Users of Unix-like operating systems, e.g., Linux and macOS, may also do bulk downloads with the program “wget” by running, as an example, the following command from the terminal:

wget -b -m -nH –cut-dirs = 6

“ftp://ftp.ebi.ac.uk/pub/databases/empiar/archive/10227/data/US1363_G1/*_tilt”

This command will retrieve all of the US1363_G1 tilt series to the directory from which the command was executed.

## Abbreviations

cryo-EM: cryo-electron microscopy/electron cryomicroscopy; cryo-ET: cryo-electron tomography/electron cryotomography; EM: electron microscopy; EMPIAR: Electron Microscopy Public Image Archive; ETDB-Caltech: Caltech Electron Tomography Database; GPU: graphical processing unit; RAM: random access memory; SPA: single-particle analysis.

## Competing interests

The authors declare that they have no competing interests.

## Funding

Singapore Ministry of Education T1 R-154-000-A49-114, T1 R-154-000-B42-114, and T2 R-154-000-B58-112.

## Authors' contributions

Experiments: C.T.N., C.C., S.C. Metadata organization and writing: L.G.

## Supplementary Material

giz077_GIGA-D-19-00104_Original_Submission

giz077_GIGA-D-19-00104_Revision_1

giz077_Response_to_Reviewer_Comments_Original_Submission

giz077_Reviewer_1_Report_Original_Submission Chris Armit -- 4/5/2019 Reviewed

giz077_Reviewer_2_Report_Original_Submission Grant Jensen -- 4/19/2019 Reviewed
