# Supplementary material for: A collection of yeast cellular electron cryotomography data
Source: Gigascience. 2019 Jun 27;8(6):giz077. doi: 10.1093/gigascience/giz077 (PMC6596884; doi:10.1093/gigascience/giz077)
Supplement: giz077_GIGA-D-19-00104_Revision_1 [file giz077_giga-d-19-00104_revision_1.pdf]

GigaScience

A collection of yeast cellular electron cryotomography data

--Manuscript Draft--

**Manuscript Number:**	GIGA-D-19-00104R1
**Full Title:**	A collection of yeast cellular electron cryotomography data
**Article Type:**	Data Note
**Funding Information:**	Ministry of Education - Singapore (R-154-000-A49-114) — Dr. Lu Gan<br>Ministry of Education - Singapore (R-154-000-B42-114) — Dr. Lu Gan<br>Ministry of Education - Singapore (R-154-000-B58-112) — Dr. Lu Gan
**Abstract:**	Background: Cells are powered by a large set of macromolecular complexes, which work together in a crowded environment. The in situ mechanisms of these complexes are unclear because their 3-D distribution, organization, and interactions are largely unknown. Electron cryotomography (cryo-ET) is a key tool to address these knowledge gaps because it produces cryotomograms – 3-D images that reveal biological structure at approximately 4-nm resolution. Cryo-ET does not involve any fixation, dehydration, staining, or plastic embedment, meaning that cellular features are visualized in a life-like, frozen-hydrated state. To study chromatin and mitotic machinery in situ, we have subjected yeast cells to a variety of genetic and/or chemical perturbations, cryosectioned them, and then imaged the cells by cryo-ET.<br><br>Findings: Every study from our group has generated more cryo-ET data than needed. Only the small subset of data that contributed to figures in these studies have been publicly shared. Here we share more than 1,000 cryo-ET raw datasets of cryosectioned budding yeast S. cerevisiae. This data will be valuable to cell biologists who are interested in the nanoscale organization of yeasts and of eukaryotic cells in general. To facilitate access, all the unpublished tilt series and a subset of corresponding cryotomograms have been deposited in the EMPIAR resource for the cell-biology community to use freely. To improve tilt series discoverability, we have uploaded metadata and preliminary notes to publicly accessible Google Sheets, EMPIAR and GigaDB.<br><br>Conclusions: Cellular cryo-ET data can be mined to obtain new cell-biological, structural, and 3-D statistical insights in situ. Because these data capture cells in a life-like state, they contain some structures that are either absent or not visible in traditional EM data. Template matching and subtomogram averaging of known macromolecular complexes can reveal their 3-D distributions and low-resolution structures. Furthermore, these data can serve as testbeds for high-throughput image-analysis pipelines, as training sets for feature-recognition software, for feasibility analysis when planning new structural cell-biology projects, and as practice data for students who are learning cellular cryo-ET.
**Corresponding Author:**	Lu Gan<br><br>SINGAPORE
**Corresponding Author Secondary Information:**	
**Corresponding Author's Institution:**	
**Corresponding Author's Secondary Institution:**	
**First Author:**	Lu Gan
**First Author Secondary Information:**	
**Order of Authors:**	Lu Gan

Powered by Editorial Manager® and ProduXion Manager® from Aries Systems Corporation

	Cai Tong Ng
	Chen Chen
	Shujun Cai
**Order of Authors Secondary Information:**	
**Response to Reviewers:**	Dear Dr. Nogoy,<br><br>Our rebuttal was formatted such that our comments were in blue indented text. However, this formatting could not be transferred to this "Respond to Reviewers" page. I hope that the Reviewers will be able to see our separate rebuttal PDF file, which is correctly formatted.<br><br>Thanks.<br>Lu<br><br><br>Dear Dr. Nogoy,<br><br>Thank you for editing our manuscript. We thank the reviewers for their positive and constructive comments. Below, we provide a point-by-point rebuttal, with the reviewers' original comments in black text and our replies in blue indented text. We have also addressed your editorial comments.<br><br>Sincerely,<br>Lu Gan<br>Editorial: I see you also have scripts in GitHub - (https://github.com/anaphaze/ot-tools) and these need an OSI (open source initiative) license assigned to them. Please choose an appropriate one and add this to GitHub.<br><br>In the repository, we have included a MIT license, which is OSI approved. This license is also included in the zipped archive that we uploaded in reply to Reviewer 1.<br><br>I have also added some minor formatting comments to your manuscript - please see the attached version and update this.<br><br>All of the suggested changes have been incorporated.<br><br>In addition, please register any new software application in the SciCrunch.org database to receive a RRID (Research Resource Identification Initiative ID) number, and include this in your manuscript. This will facilitate tracking, reproducibility and re-use of your tool.<br><br>Thank you for this suggestion. We have registered our Github ot-tools repository as RSCR_017191 and have added this RRID to the revised manuscript lines 309-311:<br><br>"Python scripts to help facilitate 3-D packing analysis of subtomograms are available in the ot-tools GitHub repository (RRID: SCR_017191) [28]."<br><br>In addition to addressing the editorial comments and reviews, we have made a few minor additions to reflect feedback on our preprint from colleagues in the field.<br><br>1) Following an interesting twitter discussion, we have added new details on how to access the EMPIAR data to lines 320-329:<br><br>"This data can be downloaded either using the Aspera Connect client or with a web browser. We do not recommend download by web browser due to its slow speed and lack of fault tolerance. Users of Unix-like operating systems, e.g., Linux and macOS, may also do bulk downloads with the program "wget" by running, as an example, the following command from the terminal:<br><br>wget -b -m -nH --cut-dirs=6<br>'ftp://ftp.ebi.ac.uk/pub/databases/empiar/archive/10227/data/US1363_G1/*_tilt'

This command will retrieve all of the US1363_G1 tilt series to the directory from which the command was executed.”

2) We have also added 3 new citations to alert readers to in situ cryo-ET papers that have explored in greater detail some of the structures presented in Figures 2 and 3:

Cytokinetic machinery: Swulius et al, PNAS 2018
Intranuclear proteasomes: Albert et al, PNAS 2017
ER-PM connection: Hoffmann et al, bioRxiv 2019

Reviewer #1: This is an excellent Data Note that uses electron cryotomography to generate 3D volumetric images that reveal organelle and macromolecular complex-level structure in the budding yeast S. cerevisiae. The dataset, composed of 1,000 cryo-ET raw datasets, is neatly organised into 5 distinct subsets that reflect the strain ID plus either the cell-cycle state or treatment. Details of pixel size are provided in the manuscript and the accompanying metadata (Google sheets), enabling measurements to be made on the volumetric images. From a reuse perspective, I was particularly pleased that the authors explored angular rotation, which is often overlooked, as there are web-based visualisation tools such as IIP3D that could allow researchers to cut arbitrary sections through these 3D data volumes. I see great reuse potential in this dataset, and I congratulate the authors for bringing attention to this in the manuscript.

Thank you for bringing to our attention the IIP3D volumetric visualization tool, which we have added to our citation list of browsing tools lin line 283-284.

From a data storage perspective, the EM images are all deposited in EMPIAR, which is the EBI resource for raw, 2D electron microscopy images. Importantly, a DOI has been ascribed to this dataset and so there is no need for the GigaScience DataBase to take a copy of the image files. The metadata are made available through Google sheets and these have allowed me to observe that these data are organised in an orderly manner that could facilitate re-use.

We thank the Reviewer for these positive comments. The sharing of surplus cellular cryo-ET data is a new phenomenon. We hope this work and the one from the Jensen lab (Ortega et al. 2019, Plos ONE) will encourage others in the field to do the same. We also hope this manuscript will stimulate feedback from the broader cell-biology community, which we can use to improve future data depositions and metadata presentation. As an example, Reviewer 2 has already suggested how anyone in the world is free to re-share our dataset as an ETDB database.

However, I do request that the authors additionally submit these metadata to the GigaScience DataBase as tabular data (comma-separated file format) to ensure long-term access.

We have now uploaded the metadata spreadsheets in .xlsx format to GigaScience. We will work with the GigaScience production staff if they require any modifications or alternative file formats. We have also uploaded a copy of the .xlsx file to EMPIAR-10227, as requested by Reviewer 2. In the revised text, each reference to the location of these metadata now also cite the copy at EMPIAR and GigaDB.

Furthermore, I suggest that a snapshot of the GutHub archive, which includes python scripts used in 3D analysis of the cryo-ET data (https://github.com/anaphaze/ot-tools), is also submitted to the GigaScience DataBase. Importantly, this GitHub archive has an OSI-approved MIT permissive free software license and therefore is open and available for reuse.

We have downloaded a copy of the GitHub archive and included it with this revision.

As a minor point, the authors make the following statement about future work:
"The current entry does not include any movie or electron-counted data. In the future, electron-counted raw data will be stored as LZW-compressed .tiff files." It would be helpful if the authors could clarify whether these data will be added to the existing EMPIAR dataset, or whether they intend on submitting these data to GigaScience.

Powered by Editorial Manager® and ProduXion Manager® from Aries Systems Corporation

	This is a good question and one that we had hoped would be resolved by now. Last year, we asked EMPIAR if we could update EMPIAR-10227 with (much) more data in the future. We were asked to wait for a decision, but have not heard back yet. We reminded EMPIAR of our question just a couple weeks ago, but unfortunately, we still have not heard back. As a compromise, we have added this sentence to lines 130-132:<br><br>"If possible, we will add the newer data to the existing entry EMPIAR-10227. Otherwise, we will create new EMPIAR entries that each contains a link to this manuscript."<br>Reviewer #2: This manuscript reports the deposition of over one thousand cryo-ET tilt-series of cryosections of budding yeast into a publicly-accessible database, EMPIAR. We agree with all the claims in the abstract, including that these tilt-series, cryotomograms, and metadata hold new information about numerous cellular structures and that these files will be useful in cryo-ET software development and training. We applaud the authors for sharing all this data with the community. The paper is well-written and clear, except for a few very minor issues (line 216). Metadata, including comments about each cryotomogram and notes about possible biological features present, are provided in public Google spreadsheets. Unfortunately many spreadsheet entries are cryptic, and there are unclear abbreviations (what do "N" and "need more dig" mean)?<br><br>Thank you for pointing on the typo in line 216. The corrected sentence reads:<br><br>"These evaluations were made from cryotomograms when possible."<br><br>We have also corrected a few other typos and strange word choices throughout the text.<br><br>The letter "N" is an abbreviation for nucleus in the "Remarks after reconstruction" and "Diagnostic remarks" columns. All of the abbreviations appear when the mouse is hovered over the google sheet notes, but we did not state this in the original manuscript. We have now pointed these notes in the revised manuscript, lines 157-160:<br><br>"During the initial annotation, we used abbreviations to denote organelles and other cellular features. These abbreviations are defined in the spreadsheet "notes", which can be displayed by hovering the mouse cursor over the spreadsheet title cell."<br><br>Phrases like "need more dig" (now removed) are either typos or personal abbreviations of the microscopists who did the initial annotation. During the preparation of this manuscript, we have spent considerable time making the annotations consistent and removing obvious typos. We are sure there are other small problems we have missed and we hope that readers will use the feedback form to bring these mistakes to our attention. We summarize these ideas in revised lines 143-145:<br><br>"The Google Sheets are "live" documents and will be updated as new datasets are deposited and as errata are brought to our attention and then corrected."<br><br>We also wonder why the authors put the metadata spreadsheets on Google rather than simply adding them to the EMPIAR folders where we think they would be more easily found.<br><br>We prefer Google Sheets because it is much easier and faster for us to curate the metadata on this resource. Nevertheless the Reviewers' question led us to the realization that Google services are blocked in some countries. To increase accessibility, we have uploaded the Excel form of the metadata to EMPIAR. We believe that in the future, we will be able to update the EMPIAR copy of the metadata, albeit with less frequency.<br><br>We agree with the authors that archiving cryo-ET datasets on resources such as EMPIAR is wonderful, but we would like to point out some advantages and disadvantages of this particular route. We've thought about this quite a bit, since we recently designed and built our own, different strategy for the same purpose (in our

Powered by Editorial Manager® and ProduXion Manager® from Aries Systems Corporation

case we shared over ten thousand tilt-series and cryotomograms of bacterial cells, see Ortega, D.R. et al. 2019. ETDB-Caltech: A blockchain-based distributed public database for electron tomography. PLOS ONE, 14, e0215531). Our image files are stored both on our servers and possibly elsewhere on a public peer-to-peer distributed file system, and our metadata is published in the FLO blockchain. Together they form a resource we named the Electron Tomography Database (ETDB). Comparing the two approaches, we believe the main advantage of the EMPIAR and Google spreadsheets mechanism used by Gan et al. is that both resources (EMPIAR and Google spreadsheets) are already familiar to researchers in the field. This familiarity will lower the access barrier. The main disadvantage is, however, that it will be clumsy for folks who want to search for and download certain diverse tilt-series, since they will have to search multiple sheets in the Google spreadsheets for what they want, then navigate the custom folder structure of these authors' special EMPIAR deposition to get them. The problem compounds if multiple groups who want to share cryotomograms upload them in similar fashion (to EMPIAR in special folder trees with spreadsheets of metadata organized in unique ways and stored in diverse places). Searches and retrievals would no longer be easily scriptable. We solved these problems, while retaining full flexibility in what metadata and data files are stored by each depositor, by using the FLO blockchain as a flexible, public, and permanent distributed ledger that serves as a universal index. The blockchain ledger system is ownerless, permissionless, and independent of grant renewals to a host institution. Additional analyses (like automatic segmentations of the tomograms by others) can be added and linked to the original data by anyone at anytime. As far as we understand, with EMPIAR and Google spreadsheets this could only be done if segmentors added their own additional google spreadsheets and published additional EMPIAR entries, again increasing the complexity of searching and finding all related material to a particular tilt-series.

We agree with every point raised here about the advantages and disadvantages of Google Sheets/EMPIAR versus ETDB. We hope that as more cryo-ET data becomes publicly available, either via EMPIAR+Google docs or ETDB, the cell biology community will use both resources and test the ideas put forth here. Perhaps an enterprising lab or individual will collect all the EMPIAR (and other publicly available) entries and create a super dataset using ETDB, as you have alluded to below.

What if ten different labs deposited cryotomography data, and then three separate software developers found ways to automatically segment cells in the cryotomograms, and each wanted to post their results linked to the original cryotomograms? Would they each have to ask all ten depositing labs permission to add their segmentation metadata to each lab's spreadsheet, or would they add their own spreadsheet referencing up to ten original spreadsheets found in potentially different places? Would they have to agree on the format of the new columns in the spreadsheet? How would the segmentations be linked to individual tomograms? ETDB's permissionless and ownerless distributed ledger (the FLO blockchain) is like a single extensible spreadsheet with metadata and permanent links to all the individual cryo-ET datasets contributed by any lab, that anyone can read and search in its entirety. Anyone can add new information to it without group debate, consensus or permission.

This is a good example of how the ownerless nature of ETDB’s metadata system allows for more flexibility in team-based annotations to a public resource.

All that being said, we view it as a great thing that different approaches are now being tried, and we note that it would be easy enough for anyone to add all of our data to EMPIAR, or add all of Gan et al.'s data to the ETDB, so time and experience will ultimately reveal which methods are best. In summary, this excellent dataset presented by the Gan Lab is a unique and valuable resource for the study of yeast ultrastructure, further development of cryo-ET software, and training.

We completely agree that these datasets can now be duplicated and therefore coexist in both the EMPIAR and ETDB systems. Our university is quite restrictive about exposing servers to the internet, so it was important for us to make our data available by a method we had already tested. Now that our data is on EMPIAR, a lab from a less-restrictive university could indeed convert EMPIAR-10227 into an ETDB database. In new lines 312 -314, we encourage this action:

	“We note that anyone can add our data to an ETDB database [14] and thereby enable the numerous benefits of ownerless-ledger metadata and decentralized storage.”
Additional Information:	
Question	Response
Are you submitting this manuscript to a special series or article collection?	No
**Experimental design and statistics**<br><br>Full details of the experimental design and statistical methods used should be given in the Methods section, as detailed in our Minimum Standards Reporting Checklist. Information essential to interpreting the data presented should be made available in the figure legends.<br><br>Have you included all the information requested in your manuscript?	Yes
**Resources**<br><br>A description of all resources used, including antibodies, cell lines, animals and software tools, with enough information to allow them to be uniquely identified, should be included in the Methods section. Authors are strongly encouraged to cite Research Resource Identifiers (RRIDs) for antibodies, model organisms and tools, where possible.<br><br>Have you included the information requested as detailed in our Minimum Standards Reporting Checklist?	Yes
**Availability of data and materials**<br><br>All datasets and code on which the conclusions of the paper rely must be either included in your submission or deposited in publicly available repositories (where available and ethically appropriate), referencing such data using a unique identifier in the references and in	Yes

Powered by Editorial Manager® and ProduXion Manager® from Aries Systems Corporation

the "Availability of Data and Materials" section of your manuscript.<br><br>Have you have met the above requirement as detailed in our Minimum Standards Reporting Checklist?	

Manuscript with DOI

Click here to view linked References

Click here to access/download;Manuscript;Gan_Surplus_data_manuscript_rev

A collection of yeast cellular electron cryotomography data

Lu Gan*, Cai Tong Ng, Chen Chen, Shujun Cai

Department of Biological Sciences and Centre for BioImaging Sciences, National University of Singapore, Singapore 117543

* Correspondence: lu@anaphase.org

ABSTRACT

Background: Cells are powered by a large set of macromolecular complexes, which work together in a crowded environment. The *in situ* mechanisms of these complexes are unclear because their 3-D distribution, organization, and interactions are largely unknown. Electron cryotomography (cryo-ET) is a key tool to address these knowledge gaps because it produces cryotomograms – 3-D images that reveal biological structure at approximately 4-nm resolution. Cryo-ET does not involve any fixation, dehydration, staining, or plastic embedment, meaning that cellular features are visualized in a life-like, frozen-hydrated state. To study chromatin and mitotic machinery *in situ*, we have subjected yeast cells to a variety of genetic and/or chemical perturbations, cryosectioned them, and then imaged the cells by cryo-ET.

Findings: Every study from our group has generated more cryo-ET data than needed. Only the small subset of data that contributed to figures in these studies have been publicly shared. Here we share more than 1,000 cryo-ET raw datasets of cryosectioned budding yeast *S. cerevisiae*. This data will be valuable to cell biologists who are interested in the nanoscale organization of yeasts and of eukaryotic cells in general. To facilitate access, all the unpublished tilt series and a subset of corresponding cryotomograms have been deposited in the EMPIAR resource for the cell-biology community to use freely. To improve tilt series discoverability, we have uploaded metadata and preliminary notes to publicly accessible Google Sheets, EMPIAR and GigaDB.

Conclusions: Cellular cryo-ET data can be mined to obtain new cell-biological, structural, and 3-D statistical insights *in situ*. Because these data capture cells in a life-like state, they contain some structures that are either absent or not visible in traditional EM data. Template matching and subtomogram averaging of known macromolecular complexes can reveal their 3-D distributions and low-resolution structures. Furthermore, these data can serve as testbeds for high-throughput image-analysis pipelines, as training sets for feature-recognition software, for feasibility analysis when planning new structural cell-biology projects, and as practice data for students who are learning cellular cryo-ET.

Keywords

yeast, chromatin, nucleus, cryo-ET, cryo-EM, template matching, mining

DATA DESCRIPTION

Background

Cryo-ET is the combination of electron cryomicroscopy (cryo-EM) and computed tomography. In a cryo-ET experiment, 2-D cryo-EM data are incrementally recorded while the sample is rotated by typical angular steps of 1° to 3° over a range of -60° to +60°. These “tilt-series” images are then mutually aligned and combined to generate a 3-D reconstruction called a cryotomogram. Because the cryotomogram contains a single field of view, cryo-ET is particularly valuable for the structural analysis of “unique” objects that cannot be averaged, such as cells [1-3]. A cryotomogram can contain a piece of tissue, a whole cell, a portion of a cell, an isolated organelle, or a field of purified macromolecular complexes. This data note focuses on cryo-ET data of cryosectioned cells.

Cryo-EM is becoming a “big data” method [4]. Highly automated cryo transmission electron microscopes, automated data-collection software, and fast-readout direct-detection cameras can now generate terabytes of data per day [5-11]. Cryo-EM “single-particle analysis” (SPA) raw data contain many copies of conformationally and constitutionally similar macromolecular complexes that are suspended in buffer. In contrast, cellular cryo-ET raw data contain many different species of macromolecular complexes. Furthermore, cellular cryo-ET data are usually recorded at lower magnification than for SPA. This dichotomy reflects (with exceptions) different goals: SPA studies aim to achieve maximum resolution of a few species of macromolecular

complexes while cellular cryo-ET studies aim to determine how macromolecular complexes are distributed and organized in their intracellular environment. SPA and cellular cryo-ET studies do share similarities. Notably, only a small percentage of the collected data contribute to the published models.

Our group has collected hundreds of tilt series per project. Because our studies are focused on one or a few types of structures, most of our data is in surplus. Two types of surplus data are “byproducts”, i.e., imaged cell positions that lack the targeted structures, and “bystanders”, i.e., imaged cellular structures nearby to the targeted structures. We have previously shared cryo-ET data with collaborators and colleagues using commercial internet solutions like Dropbox and Google Drive, but we found that these tools were suboptimal for sharing multi-gigabyte files. Alternative web technologies have allowed resources such as Electron Microscopy Public Image Archive (EMPIAR) [12] and the Caltech Electron Tomography Database (ETDB-Caltech) [13, 14] to share terabyte-sized datasets globally and more conveniently. We have deposited our published and surplus cryo-ET tilt series data in EMPIAR.

Context

We are interested in the relationship between macromolecular structure and function inside cell nuclei. As a model system, we use yeast cells that are arrested at well-defined points in the cell cycle (Fig. 1A). We have shown that chromatin is packed irregularly, without forming any monolithic condensed structures in both interphase and

mitosis [15, 16] and that the majority of outer-kinetochore Dam1C/DASH complexes assembles as partial rings and does not contact the kinetochore microtubules' curved tips *in situ* [17]. These studies show that the intracellular distribution and organization of macromolecular complexes are not always consistent with the models derived from *in vitro* studies. Indeed, our efforts to locate Dam1C/DASH *in situ* were hampered because we originally searched for complete rings resting against curved microtubule protofilaments. We also had difficulty locating condensed chromosomes in fission yeast because we were expecting to find a monolithic nucleosome mass separated from a relatively "empty" nucleoplasm.

Our group has recorded more than one thousand tilt series of cryosectioned yeast cells. These include the budding yeast *Saccharomyces cerevisiae* and the fission yeast *Schizosaccharomyces pombe*. Only a minority of our tilt series were presented in a paper; this data subset is already available at EMPIAR. Here we present the surplus cellular tilt series data we collected as part of those published studies. We have neither analyzed nor intend to analyze in detail the vast majority of this data. These data will be valuable to other groups interested in macromolecular complexes and cytological features both within and outside the nucleus. Because the typical cryotomogram has ~ 4-nm resolution, many structures can be identified on the basis of their shape, size, and intracellular context. The cryotomographic densities of some of these structures may contain features that are difficult to see in EM images of plastic sections. Notable examples are nucleosomes and some of the smaller or thinner components of the

chromosome segregation and cell-division machineries.

Dataset format and logistics

All cryo-ET data files are saved in the MRC format [18] under the accession code EMPIAR-10227 (Fig. 1B). Each dataset has a unique name that combines the date of data collection and a serial number. For example, 18jun04a__02 is the second tilt series collected on 2018, June 4, session “a”. Future depositions may use the alternative YYYYMMDD_SN format, making the previous example 20180604_02. Some datasets include both a tilt series and a cryotomogram. The filename extensions follow IMOD conventions: “.mrc” for tilt series and “.rec” for cryotomograms. To conserve storage space and speed up file transfers, the tilt series and cryotomograms have been compressed with lbzip2, and therefore have the “.bz2” extension. The current entry does not include any movie or electron-counted data. In the future, electron-counted raw data will be stored as LZW-compressed .tiff files. If possible, we will add the newer data to the existing entry EMPIAR-10227. Otherwise, we will create new EMPIAR entries that each contains a link to this manuscript.

The pixel sizes used in the present data range from 4.6 to 9.1 Ångstroms. Most of the data were recorded on direct-detection cameras that have ~ 16 million pixels in a 4,096 x 4,096 pixel array. The typical field of view therefore ranges from ~ 2 to 4 µm squared. Most tilt series consist of approximately 61 images because we typically use a ±60° tilt range and a 2° tilt increment. The pixel intensity values in most tilt-series data are stored as 16-bit unsigned integers, so the typical tilt series is ~ 2 gigabytes.

We have shared via Google Sheets [19], EMPIAR [20], and GigaDB [21] a set of tabbed spreadsheets that contain metadata and preliminary notes and observations (Fig. 1C, also see Availability section). The Google Sheets are “live” documents and will be updated as new datasets are deposited and as errata are brought to our attention and then corrected. The first spreadsheet tab has a summary of all the data, links to additional related resources, commonly used commands, and a link to an online feedback form. Subsequent spreadsheet tabs contain detailed information on each tilt series, grouped by a strain ID and a treatment condition. For example, the “US1363_nocodazole” spreadsheet describes cryo-ET data of US1363 cells that were treated with the tubulin-polymerization inhibitor nocodazole.

In the detailed metadata spreadsheets, each row corresponds to one tilt series. Some cells were imaged by serial cryo-ET and therefore have the sequence number of each contributing tilt series noted in the “S/N” column. The other columns organize the data-collection parameters, appraisal of image contrast, diagnostic remarks on the data-collection session and quality, and a guess about the cytological features and macromolecular complexes present in the imaged cell. During the initial annotation, we used abbreviations to denote organelles and other cellular features. These abbreviations are defined in the spreadsheet “notes”, which can be displayed by hovering the mouse cursor over the spreadsheet title cell. The accuracy of some of our annotations of cytological features is limited by our current cryo-ET and cell-biology knowledge, but will improve with both experience and especially user feedback. We

anticipate that cell biologists will use the sorting function to shortlist the tilt series most salient to their studies.

Methods

Cells were either grown in conditions that arrest populations at defined stages of the cell cycle or treated with drugs to perturb their cytology and cell-cycle progress. Because of our interest in mitosis and chromosome condensation, the present data capture cells in G1 phase, metaphase, and in mitosis with disrupted mitotic spindles. Liquid-cultured cells were collected by either centrifugation or vacuum filtration. These cells were then either high-pressure frozen or self-pressurized frozen in the presence of the extracellular cryoprotectant dextran. The frozen-hydrated cell block was sectioned in a cryomicrotome, producing a ribbon of cryosections. This cryosection ribbon was attached to either a continuous- or holey-carbon EM grid, which had been pre-coated with 10-nm-diameter gold nanoparticles. Nanoparticles serve as easy-to-locate fiducial markers that facilitate tilt-series alignment. The cell cryosections were then imaged on a Titan Krios equipped with a direct detector, with or without Volta phase contrast. Additional details can be found in our earlier papers [15-17] and in the online spreadsheets.

Cryotomogram reconstruction, visualization, and analysis were done on a modern workstation computer with popular open-source software (Table 1). Radiation damage causes some cryosection positions to undergo non-uniform distortions, meaning that alignments were done using only the fiducials proximal to the structure of interest. Most

of the tilt series were aligned using 4 to 12 fiducials coincident with the nuclei. To improve the visualization of other features, users should do a "local" alignment using only the fiducial markers closer to their structures of interest. If local alignment is not desired, the tilt series can be semi-automatically aligned using fiducials spread throughout the field of view and then reconstructed using software like Etomo and Protomo [22, 23]. Such cryotomograms tend to have uniform resolution at all positions where the cryosection is in contact with the carbon substrate.

Cryotomograms are noisier than SPA reconstructions, meaning that these datasets are very difficult to comprehend when visualized as isosurfaces. Instead, cryotomograms should be visualized as cryotomographic slices: 2-D images that average multiple voxels along one axis. The slice thickness should match the structure of interest, e.g., 10 nm for nucleosomes. To facilitate comparison between datasets, multiple cryotomograms can be simultaneously loaded into random-access memory in one instance of the program 3dmod [22]. Assuming they all "fit" into memory, cryotomograms loaded this way can be rapidly toggled in sequence using the "1" and "2" shortcut keys.

Reconstructed cryotomograms are usually the starting point of more quantitative analysis. Examples of deeper analysis by template matching, classification, and subtomogram averaging can be found in recent reviews and the many excellent papers cited within [24-27]. Because structural cell biology is a new field, most of our studies have required new analysis tools. We have written a number of python scripts to

facilitate the 3-D packing analysis of subtomograms [28]. These scripts control programs from published image-analysis packages [22, 29-31], most of which are open source.

Data validation and quality control

The yeast cryo-ET data have been recorded under a variety of conditions (magnification, dose, tilt increment, defocus) with different contrast mechanisms (defocus phase contrast vs. Volta phase contrast). Furthermore, the tilt series have differences in quality due to variations in freezing, attachment to the grid, radiation damage, or a combination of these factors. Owing to this variability, we cannot assign a single validation metric to the entire set of tilt series. We have qualitatively assessed each tilt series' contrast relative to others recorded in the same session (tens of tilt series per session). The contrast is rated from one to five stars and is recorded in the online spreadsheet columns marked with the "◐ " symbol. Four- to five-star data typically reveal features like membrane leaflets, clear separation of nucleosome-like particles, and particles smaller than nucleosomes. These evaluations were made from cryotomograms when possible.

The deposited cryotomograms should be considered preliminary for three reasons. First, most of the cryotomograms were reconstructed using the subset of fiducial markers coincident with the nucleus, which results in lower reconstruction quality elsewhere in the cell. Second, the fiducial centers were manually fine-tuned for the few tilt series that contributed to the final published figures. Third, we anticipate that future

developments in fiducial-assisted and fiducial-less alignment will produce better cryotomograms than currently possible.

Re-use potential

The deposited yeast cryo-ET data contain a large number easy-to-find or abundant organelles and macromolecular complexes such as mitochondria, eisosomes, cytokinetic machinery, microtubule-organizing centers, fatty acid synthases, proteasomes, vacuoles, rough endoplasmic reticulum, lipid bodies, and cytoplasmic amorphous aggregates (Fig. 2). Closer inspection may reveal poorly documented subcellular features. Examples of such features include mitochondrial internal filaments (Fig. 2A) and ordered layers in lipid-droplet-like bodies (Fig. 2I). Cell biologists may want use these data to measure local concentrations of macromolecular complexes, detect interactions between these complexes, determine the orientations of large complexes *in situ*, test for the existence of putative cellular features, and determine how cellular bodies make direct contact with one another. Furthermore, this data will provide morphological, distance, or stoichiometric constraints for researchers that are attempting to reconstitute either a complex or a cellular body.

If multiple copies of a macromolecular complex can be detected in one or more cellular cryotomograms, they can be computationally extracted and then analyzed as “single particles”. In this subtomogram-averaging approach, the subtomograms are aligned and then averaged together to create density maps that have higher-resolution features visible, as discussed in recent reviews [24-27]. The centers of mass and orientation

information can then be used to remap the average back into a volume the same size as the cryotomogram. If the complexes are densely packed, these remapped models will reveal higher-order structure as seen in polysomes and oligonucleosomes [32, 33].

Cellular cryotomograms also contain hard-to-find structures (Fig. 3). These structures are either rare or are located in cellular positions that we rarely target, such as the bud neck (Fig. 3C). Many of these structures, such as inter-membrane contact sites (Fig 3F) and lipid-body protrusions (Fig. 3H) are poorly documented in the cryo-ET literature. We anticipate that yeast cryo-ET data will help stimulate the discovery and detailed characterization of interesting eukaryotic subcellular bodies just as cellular cryo-ET has done for bacterial cell biology [34-38]. Furthermore, structures that are identified by other groups can be retrospectively analyzed in this data in the context of known cell-cycle states and pharmacological perturbations.

Users should note we arrested the yeast cells in various cell-cycle stages to allow comparative studies of nuclear structures like chromatin, spindles, and kinetochores. Because the cell cycle affects the entire proteome, this dataset will shed light on how other organelles and cytoplasmic macromolecular complexes are cell-cycle regulated. Some of the structures observed in this yeast data may also be stress-induced. Indeed, recent studies showed that upon starvation, eukaryotic translation initiation factor 2B forms large filament bundles in budding yeast [39, 40].

These data span a range of defoci and magnifications, with or without the Volta phase contrast [41]. Such experimental diversity will allow software developers to test the robustness of new image-processing routines used in automated alignment [22, 23], template matching (also called 3-D particle picking), subtomogram averaging and classification [31, 42-45]. The yeast cryo-ET data can also be used to train machine-learning algorithms to detect features in both tilt series and cryotomograms [46-48]. Furthermore, data-sharing resources may use this data to develop annotation and browsing tools [49-51].

The vast majority of our cryo-ET imaging was recorded with first generation direction-detection cameras, without energy filtering. If either the structure of interest or a structure of equivalent size can be detected in the present data, then it will most certainly be detectable in data recorded on electron-counting cameras, both with or without energy filtering. Therefore, these data will facilitate feasibility analyses.

Finally, new structural cell biologists will find these data useful as real-world examples that complement the lessons from cryo-EM tutorials [52, 53]. The vast majority of the deposited data are from grids that have gold nanoparticles, making the alignment process similar to – and therefore a direct follow-on to the IMOD plastic-section tutorial dataset [53]. Students can use the reconstructed cryotomograms to practice manual annotation and more automated analyses such as template matching and subtomogram averaging.

Availability of supporting data

We have deposited our data under accession code EMPIAR-10227 [20]. We excluded "unusable" tilt series, which have one or more of the following image or sample properties: extreme drift, occlusion by large ice crystals, cracks in the ice or carbon substrate, or completely detached sections. We also included a copy of the tilt series that were already deposited as part of our original research papers. Key metadata are available in Google Sheets [19] and EMPIAR [20], which can be copied to the user's own Google Drive or downloaded as a Microsoft Excel spreadsheet file. Thereafter, the user can sort the rows to identify smaller subsets of tilt series that have the desired properties or structures. Python scripts to help facilitate 3-D packing analysis of subtomograms are available in the ot-tools GitHub repository (RRID: SCR_017191) [28]. A copy of the metadata spreadsheets and the ot-tools scripts are available in the *GigaScience* database, GigaDB [21]. We note that anyone can add our data to an ETDB database [14] and thereby enable the numerous benefits of ownerless-ledger metadata and decentralized storage.

Feedback can be sent via a Google Form [54].

The tilt series and cryotomograms are organized in the following directory structure:

```
Sample_ID_1
      Session_ID
            Tilt_series
                  series_first.mrc.bz2
```

```
                    ...
                    series_last.mrc.bz2
              Tomograms
                    series_first.rec.bz2
                    ...
                    series_last.rec.bz2
Sample_ID_2
       Session_ID
              Tilt_series
                    series_first.mrc.bz2
                    ...
                    series_last.mrc.bz2
              Tomograms
                    series_first.rec.bz2
                    ...
                    series_last.rec.bz2
```

This data can be downloaded either using the Aspera Connect client or with a web browser. We do not recommend download by web browser due to its slow speed and lack of fault tolerance. Users of Unix-like operating systems, e.g., Linux and macOS, may also do bulk downloads with the program “wget” by running, as an example, the following command from the terminal:

```
wget -b -m -nH --cut-dirs=6
'ftp://ftp.ebi.ac.uk/pub/databases/empiar/archive/10227/data/US1363_G1/*_tilt
'
```

This command will retrieve all of the US1363_G1 tilt series to the directory from which the command was executed.

Abbreviations

cryo-EM: cryo-electron microscopy / electron cryomicroscopy; cryo-ET: cryo-electron tomography / electron cryotomography

Competing interests

The authors do not have any competing interests.

Funding

Singapore Ministry of Education T1 R-154-000-A49-114, T1 R-154-000-B42-114, and T2 R-154-000-B58-112.

Authors' contributions

Experiments: CTN, CC, SC. Metadata organization and writing: LG.

Acknowledgements

We thank Gemma An and Chithran VM for help with some reconstructions; Uttam Surana and Mohan Balasubramanian for the yeast strains; Ardan Patwardhan and Andrii Iudin for feedback on data organization; Ben Himes for suggestions of additional download options; Christoph Baranec for discussion on astronomy data-sharing practices; Paul Matsudaira, Jian Shi, Ann Tran, and Ping Lee Chong for setting up and

operating the cryo-EM platform at the National University of Singapore Centre for BioImaging Sciences; and our many colleagues for discussions on interesting cell-biology questions.

REFERENCES

1. Oikonomou CM and Jensen GJ. Cellular Electron Cryotomography: Toward Structural Biology In Situ. Annu Rev Biochem. 2017;86:873-96. doi:10.1146/annurev-biochem-061516-044741.

2. Pfeffer S and Mahamid J. Unravelling molecular complexity in structural cell biology. Curr Opin Struct Biol. 2018;52:111-8. doi:10.1016/j.sbi.2018.08.009.

3. Weber MS, Wojtynek M and Medalia O. Cellular and Structural Studies of Eukaryotic Cells by Cryo-Electron Tomography. Cells. 2019;8 1 doi:10.3390/cells8010057.

4. Baldwin PR, Tan YZ, Eng ET, Rice WJ, Noble AJ, Negro CJ, et al. Big data in cryoEM: automated collection, processing and accessibility of EM data. Curr Opin Microbiol. 2018;43:1-8. doi:10.1016/j.mib.2017.10.005.

5. Mastronarde DN. Automated electron microscope tomography using robust prediction of specimen movements. J Struct Biol. 2005;152 1:36-51. doi:10.1016/j.jsb.2005.07.007.

6. Suloway C, Shi J, Cheng A, Pulokas J, Carragher B, Potter CS, et al. Fully automated, sequential tilt-series acquisition with Leginon. J Struct Biol. 2009;167 1:11-8. doi:10.1016/j.jsb.2009.03.019.

7. Lander GC, Stagg SM, Voss NR, Cheng A, Fellmann D, Pulokas J, et al. Appion: an integrated, database-driven pipeline to facilitate EM image processing. J Struct Biol. 2009;166 1:95-102.

8. Tan YZ, Cheng A, Potter CS and Carragher B. Automated data collection in single particle electron microscopy. Microscopy (Oxf). 2016;65 1:43-56. doi:10.1093/jmicro/dfv369.

9. McMullan G, Faruqi AR, Henderson R, Guerrini N, Turchetta R, Jacobs A, et al. Experimental observation of the improvement in MTF from backthinning a CMOS direct electron detector. Ultramicroscopy. 2009;109 9:1144-7. doi:10.1016/j.ultramic.2009.05.005.

10. Milazzo AC, Moldovan G, Lanman J, Jin L, Bouwer JC, Klienfelder S, et al. Characterization of a direct detection device imaging camera for transmission electron microscopy. Ultramicroscopy. 2010;110 7:744-7. doi:10.1016/j.ultramic.2010.03.007.

11. Li X, Mooney P, Zheng S, Booth CR, Braunfeld MB, Gubbens S, et al. Electron counting and beam-induced motion correction enable near-atomic-resolution single-particle cryo-EM. Nat Methods. 2013;10 6:584-90. doi:10.1038/nmeth.2472.

12. Iudin A, Korir PK, Salavert-Torres J, Kleywegt GJ and Patwardhan A. EMPIAR: a public archive for raw electron microscopy image data. Nat Methods. 2016;13 5:387-8. doi:10.1038/nmeth.3806.

13. Ding HJ, Oikonomou CM and Jensen GJ. The Caltech Tomography Database and Automatic Processing Pipeline. J Struct Biol. 2015;192 2:279-86. doi:10.1016/j.jsb.2015.06.016.

14. Ortega DR, Oikonomou CM, Ding HJ, Rees-Lee P, Alexandria and Jensen GJ. ETDB-Caltech: A blockchain-based distributed public database for electron tomography. PLoS One. 2019;14 4:e0215531. doi:10.1371/journal.pone.0215531.

15. Chen C, Lim HH, Shi J, Tamura S, Maeshima K, Surana U, et al. Budding yeast chromatin is dispersed in a crowded nucleoplasm in vivo. Mol Biol Cell. 2016;27 21:3357-68. doi:10.1091/mbc.E16-07-0506.

16. Cai S, Chen C, Tan ZY, Huang Y, Shi J and Gan L. Cryo-ET reveals the macromolecular reorganization of S. pombe mitotic chromosomes in vivo. Proc Natl Acad Sci U S A. 2018;115 43:10977-82. doi:10.1073/pnas.1720476115.

17. Ng CT, Deng L, Chen C, Lim HH, Shi J, Surana U, et al. Electron cryotomography analysis of Dam1C/DASH at the kinetochore-spindle interface in situ. J Cell Biol. 2019;218 2:455–73. doi:10.1083/jcb.201809088.

18. Cheng A, Henderson R, Mastronarde D, Ludtke SJ, Schoenmakers RH, Short J, et al. MRC2014: Extensions to the MRC format header for electron cryo-microscopy and tomography. J Struct Biol. 2015;192 2:146-50. doi:10.1016/j.jsb.2015.04.002.

19. Gan L, Ng CT, Chen C and Cai S: A collection of yeast cellular electron cryotomography data - Google Sheets metadata. https://goo.gl/mwWyTk (2019).

20. Gan L, Ng CT, Chen C and Cai S: A collection of yeast cellular electron cryotomography data - EMPIAR-10227. http://www.ebi.ac.uk/pdbe/emdb/empiar/entry/10227/ (2018).

21. Gan L; Ng CT; Chen C; Cai S: Supporting data for "A collection of yeast cellular electron cryotomography data" GigaScience Database. 2019. http://dx.doi.org/10.5524/100609

22. Mastronarde DN. Dual-axis tomography: an approach with alignment methods that preserve resolution. J Struct Biol. 1997;120 3:343-52. doi:10.1006/jsbi.1997.3919.

23. Noble AJ and Stagg SM. Automated batch fiducial-less tilt-series alignment in Appion using Protomo. J Struct Biol. 2015;192 2:270-8. doi:10.1016/j.jsb.2015.10.003.

24. Rossmann FM and Beeby M. Insights into the evolution of bacterial flagellar motors from high-throughput in situ electron cryotomography and subtomogram averaging. Acta Crystallogr D Struct Biol. 2018;74 Pt 6:585-94. doi:10.1107/S2059798318007945.

25. Hutchings J and Zanetti G. Fine details in complex environments: the power of cryo-electron tomography. Biochem Soc Trans. 2018;46 4:807-16. doi:10.1042/BST20170351.

26. Wan W and Briggs JA. Cryo-Electron Tomography and Subtomogram Averaging. Methods Enzymol. 2016;579:329-67. doi:10.1016/bs.mie.2016.04.014.

27. Asano S, Engel BD and Baumeister W. In Situ Cryo-Electron Tomography: A Post-Reductionist Approach to Structural Biology. J Mol Biol. 2016;428 2 Pt A:332-43. doi:10.1016/j.jmb.2015.09.030.

28. Gan L: ot-tools. https://github.com/anaphaze/ot-tools (2018). Accessed May 1 2019.

29. Heymann JB and Belnap DM. Bsoft: image processing and molecular modeling for electron microscopy. J Struct Biol. 2007;157 1:3-18. doi:10.1016/j.jsb.2006.06.006.

30. Tang G, Peng L, Baldwin PR, Mann DS, Jiang W, Rees I, et al. EMAN2: an extensible image processing suite for electron microscopy. J Struct Biol. 2007;157 1:38-46. doi:10.1016/j.jsb.2006.05.009.

31. Bharat TA, Russo CJ, Lowe J, Passmore LA and Scheres SH. Advances in Single-Particle Electron Cryomicroscopy Structure Determination applied to Sub-tomogram Averaging. Structure. 2015;23 9:1743-53. doi:10.1016/j.str.2015.06.026.

32. Mahamid J, Pfeffer S, Schaffer M, Villa E, Danev R, Cuellar LK, et al. Visualizing the molecular sociology at the HeLa cell nuclear periphery. Science. 2016;351 6276:969-72. doi:10.1126/science.aad8857.

33. Cai S, Böck D, Pilhofer M and Gan L. The in situ structures of mono-, di-, and trinucleosomes in human heterochromatin. Mol Biol Cell. 2018;29 20:2450-7. doi:10.1091/mbc.E18-05-0331.

34. Briegel A, Dias DP, Li Z, Jensen RB, Frangakis AS and Jensen GJ. Multiple large filament bundles observed in Caulobacter crescentus by electron cryotomography. Mol Microbiol. 2006;62 1:5-14. doi:10.1111/j.1365-2958.2006.05355.x.

35. Dobro MJ, Oikonomou CM, Piper A, Cohen J, Guo K, Jensen T, et al. Uncharacterized bacterial structures revealed by electron cryotomography. J Bacteriol. 2017; doi:10.1128/JB.00100-17.

36. Ingerson-Mahar M, Briegel A, Werner JN, Jensen GJ and Gitai Z. The metabolic enzyme CTP synthase forms cytoskeletal filaments. Nat Cell Biol. 2010;12 8:739-46. doi:10.1038/ncb2087.

37. Swulius MT, Chen S, Jane Ding H, Li Z, Briegel A, Pilhofer M, et al. Long helical filaments are not seen encircling cells in electron cryotomograms of rod-shaped bacteria. Biochem Biophys Res Commun. 2011;407 4:650-5. doi:10.1016/j.bbrc.2011.03.062.

38. Basler M, Pilhofer M, Henderson GP, Jensen GJ and Mekalanos JJ. Type VI secretion requires a dynamic contractile phage tail-like structure. Nature. 2012;483 7388:182-6. doi:10.1038/nature10846.

39. Marini G, Nueske E, Leng W, Alberti S and Pigino G. Adaptive reorganization of the cytoplasm upon stress in budding yeast. bioRxiv. 2018.

40. Nueske E, Marini G, Richter D, Leng W, Bogdanova A, Franzmann TM, et al. Filament formation by the translation factor eIF2B regulates protein synthesis in starved cells. bioRxiv. 2018.

41. Fukuda Y, Laugks U, Lucic V, Baumeister W and Danev R. Electron cryotomography of vitrified cells with a Volta phase plate. J Struct Biol. 2015;190 2:143-54. doi:10.1016/j.jsb.2015.03.004.

42. Nicastro D, Schwartz C, Pierson J, Gaudette R, Porter ME and McIntosh JR. The molecular architecture of axonemes revealed by cryoelectron tomography. Science. 2006;313 5789:944-8. doi:10.1126/science.1128618.

43. Heumann JM: PEET. http://bio3d.colorado.edu/PEET/ (2016). Accessed March 22 2017.

44. Forster F, Han BG and Beck M. Visual proteomics. Methods Enzymol. 2010;483:215-43. doi:10.1016/S0076-6879(10)83011-3.

45. Castano-Diez D, Kudryashev M and Stahlberg H. Dynamo Catalogue: Geometrical tools and data management for particle picking in subtomogram averaging of cryo-electron tomograms. J Struct Biol. 2017;197 2:135-44. doi:10.1016/j.jsb.2016.06.005.

46. Chen M, Dai W, Sun SY, Jonasch D, He CY, Schmid MF, et al. Convolutional neural networks for automated annotation of cellular cryo-electron tomograms. Nat Methods. 2017;14 10:983-5. doi:10.1038/nmeth.4405.

47. Zeng X, Leung MR, Zeev-Ben-Mordehai T and Xu M. A convolutional autoencoder approach for mining features in cellular electron cryo-tomograms and weakly supervised coarse segmentation. J Struct Biol. 2018;202 2:150-60. doi:10.1016/j.jsb.2017.12.015.

48. Xu M, Singla J, Tocheva EI, Chang YW, Stevens RC, Jensen GJ, et al. De Novo Structural Pattern Mining in Cellular Electron Cryotomograms. Structure. 2019; doi:10.1016/j.str.2019.01.005.

49. Husz ZL, Burton N, Hill B, Milyaev N and Baldock RA. Web tools for large-scale 3D biological images and atlases. BMC Bioinformatics. 2012;13:122. doi:10.1186/1471-2105-13-122.

50. Patwardhan A, Ashton A, Brandt R, Butcher S, Carzaniga R, Chiu W, et al. A 3D cellular context for the macromolecular world. Nat Struct Mol Biol. 2014;21 10:841-5. doi:10.1038/nsmb.2897.

51. Abbott S, Iudin A, Korir PK, Somasundharam S and Patwardhan A. EMDB Web Resources. Curr Protoc Bioinformatics. 2018;61 1:5 10 1-5 2. doi:10.1002/cpbi.48.

52. Vos MR and Jensen GJ: Getting Started in Cryo-EM online course. https://em-learning.com/ (2018). Accessed November 21 2018.

53. O'Toole E: ETomo Tutorial for IMOD Version 4.9. https://bio3d.colorado.edu/imod/doc/etomoTutorial.html (2018). Accessed January 1 2018.

54. Gan L, Ng CT, Chen C and Cai S: A collection of yeast cellular electron cryotomography data - Google Forms feedback. https://goo.gl/forms/FtU8RtbXCfbAa2gn2 (2019).

55. Bharat TAM, Hoffmann PC and Kukulski W. Correlative Microscopy of Vitreous Sections Provides Insights into BAR-Domain Organization In Situ. Structure. 2018;26 6:879-86 e3. doi:10.1016/j.str.2018.03.015.

56. Swulius MT, Nguyen LT, Ladinsky MS, Ortega DR, Aich S, Mishra M, et al. Structure of the fission yeast actomyosin ring during constriction. Proc Natl Acad Sci U S A. 2018;115 7:E1455-E64. doi:10.1073/pnas.1711218115.

57. Albert S, Schaffer M, Beck F, Mosalaganti S, Asano S, Thomas HF, et al. Proteasomes tether to two distinct sites at the nuclear pore complex. Proc Natl Acad Sci U S A. 2017;114 52:13726-31. doi:10.1073/pnas.1716305114.

58. Hoffmann PC, Bharat TAM, Wozny MR, Miller EA and Kukulski W. Rod-shaped tricalbins contribute to PM asymmetry at curved ER-PM contact sites. bioRxiv. 2019.

Table 1: Recommended hardware and software

Tool	Recommendation	Notes
Computer	Modern workstation	More memory (RAM) facilitates comparisons of multiple cryotomograms.
Display	27+ inch monitor	
Operating system	Linux	Most cryo-EM software is developed on Linux; extra effort is needed to run this software in macOS or Windows.
Visualization software	3dmod (IMOD)	FIJI can also be used, but it is not optimized for tomography data.
Reconstruction software	Etomo (IMOD)	A solid-state disk and a CUDA-compatible NVIDIA GPU are highly recommended.
Download client	Aspera Connect	This software enables fast, fault-tolerant downloads from EMPIAR.
Notes	Google Sheets, Microsoft Excel	The shared spreadsheet can be downloaded and then customized.

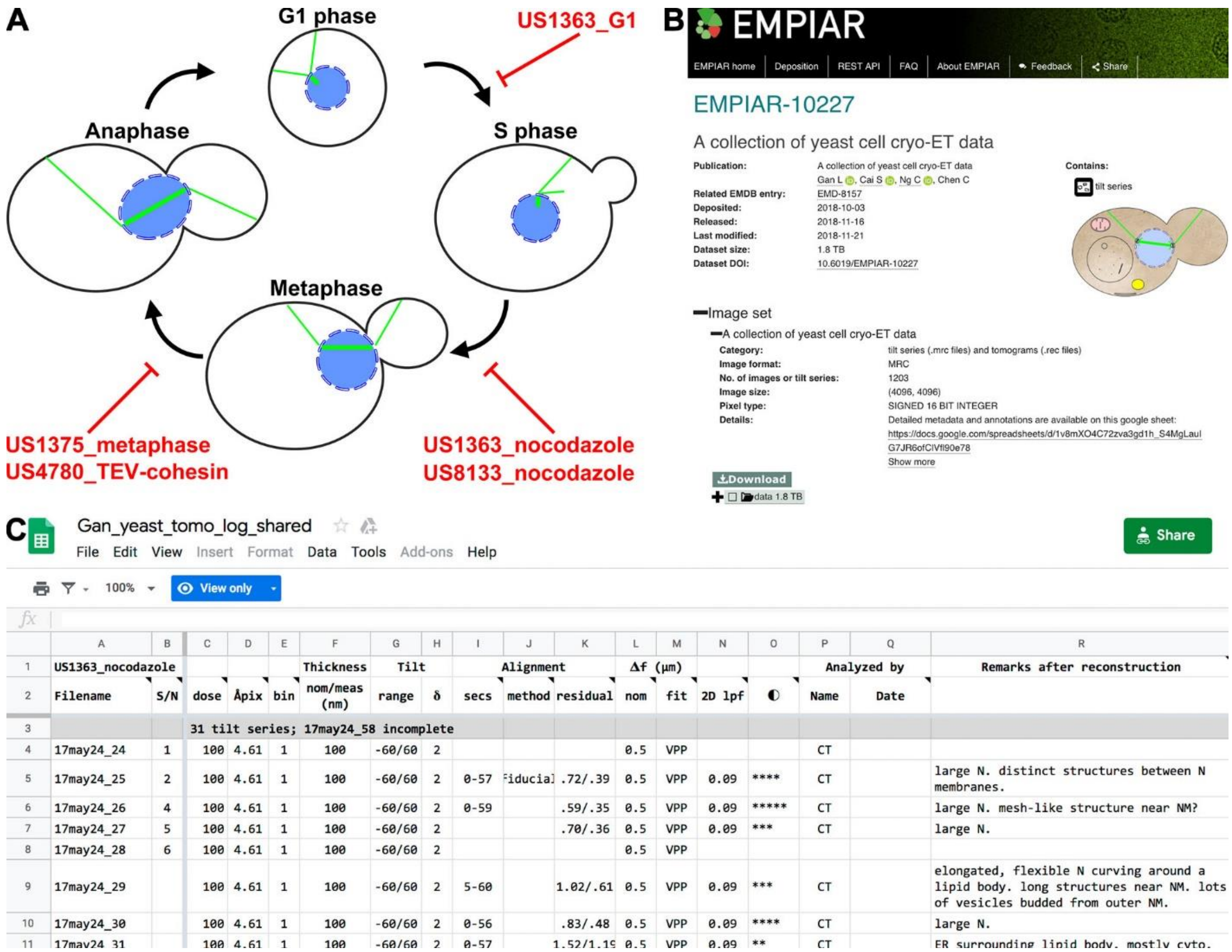

Figure 1: Yeast cryo-ET dataset summary

Yeast cell-cycle stages sampled by this data. The red text indicates the strain ID plus either the cell-cycle state or treatment. **(B)** Screenshot of the EMPIAR entry. Downloads are faster and more reliable when done with the recommended client (Aspera Connect, as of this writing). **(C)** Screenshot of the preliminary notes, which are shared in Google Sheets tabs named after the red text in panel A. The metadata are also available from EMPIAR [20] and GigaDB [21] as a Microsoft Excel spreadsheet file.

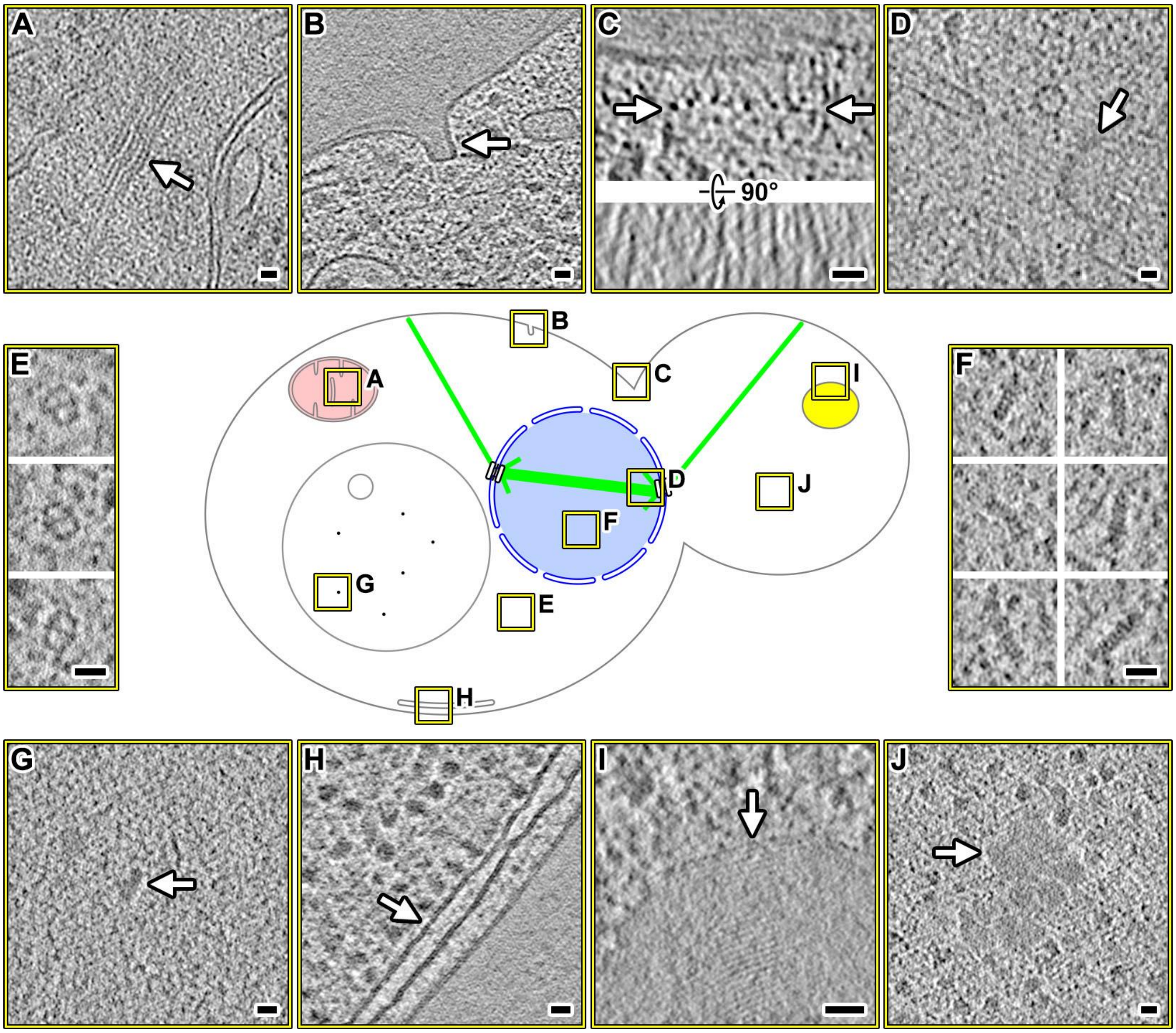

Figure 2: Easy-to-find structures in yeast cryotomograms

Center: graphical legend showing the locations of interesting features (boxed in yellow), which are enlarged as cryotomographic slices (10 - 20 nm thick). **(A)** Filament bundle within a mitochondrion. **(B)** Eisosome; see [55] for identification details. **(C)** Cytokinetic machinery. Upper panel: transverse view. The row of filamentous complexes is indicated by arrows. Lower panel: longitudinal view of the filaments. See [56] for examples of fission-yeast cytokinetic machinery. **(D)** Microtubule-organizing center. **(E)** Fatty acid synthases. **(F)** Intranuclear proteasomes; see [57] for examples of algal

intranuclear proteasomes. **(G)** Particles inside a vacuole. **(H)** Endoplasmic reticulum adjacent to the plasma membrane. **(I)** Lipid-droplet-like body with periodic internal structure. **(J)** Amorphous cytoplasmic aggregate. Scale bar = 20 nm in all panels.

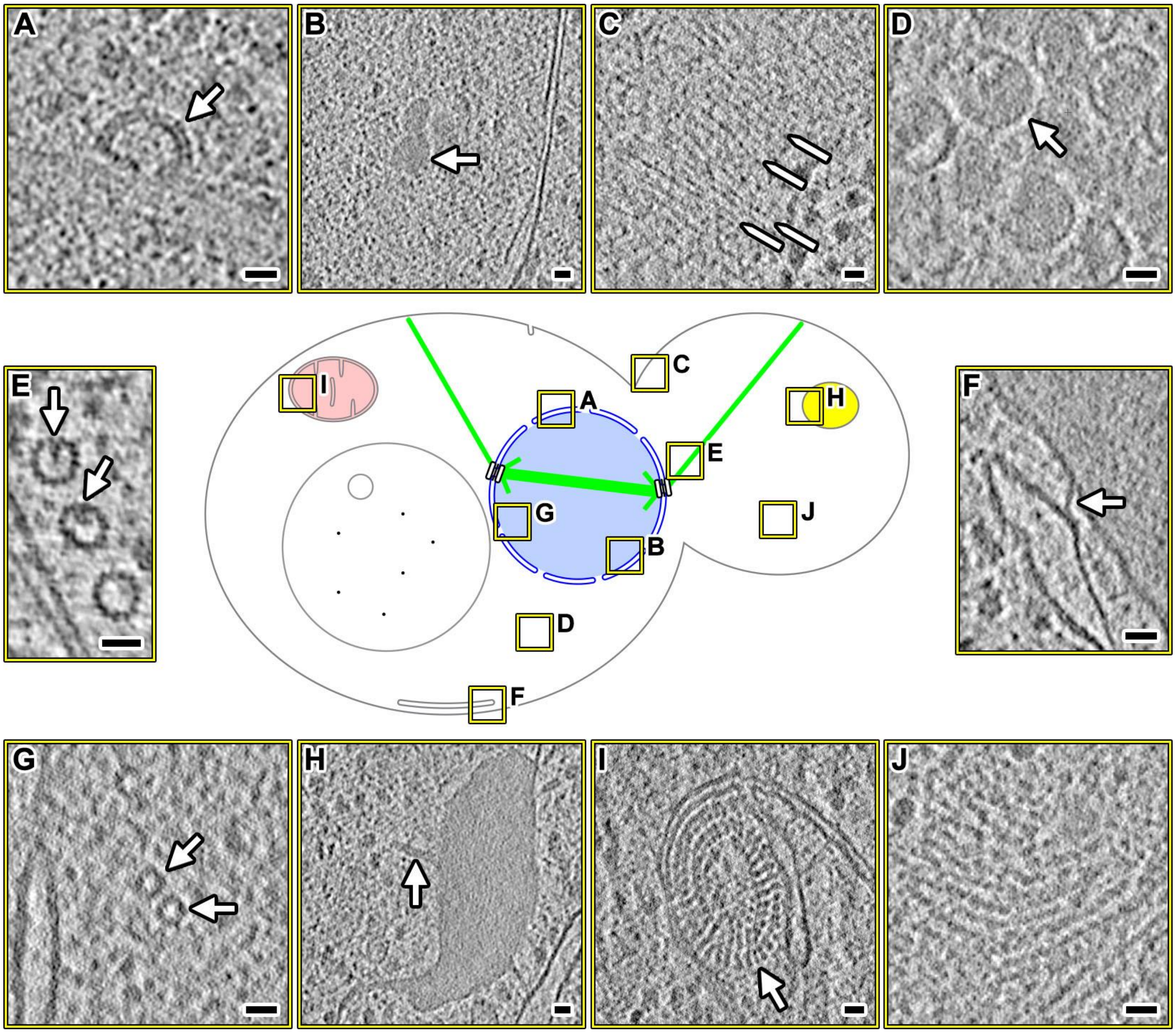

Figure 3: Hard-to-find structures in yeast cryotomograms

Center: graphical legend showing the locations of interesting features (boxed in yellow), which are enlarged as cryotomographic slices (10 - 20 nm thick). **(A)** A coated pit-like structure, docked to the outer nuclear membrane. **(B)** Intranuclear granule. **(C)** Septin-like cytokinesis machinery. A few examples are indicated by the pointed lines. These filaments run parallel to the mother-daughter cell axis. **(D)** Virus-like particles in the cytoplasm. **(E)** Luminal particles in cytoplasmic microtubules. **(F)** Connection between the endoplasmic reticulum and plasma membrane; see [58] for more details about these

inter-membrane connections. **(G)** Short intranuclear 15-nm diameter tubes. **(H)** A lipid body with thin protrusions, one of which is indicated by the arrow. **(I)** Mitochondrial periodic structures extending from the inner membrane into the matrix. **(J)** Filamentous cytoplasmic aggregates.

Metadata spreadsheets

Click here to access/download
Supplementary Material
Gan_yeast_tomo_log_shared.xlsx

ot-tools license

Click here to access/download
Supplementary Material
LICENSE

ot-tools readme

Click here to access/download

Supplementary Material

README.md

ot-tools script

Click here to access/download
Supplementary Material
nearestneighbour.m

ot-tools script

Click here to access/download
Supplementary Material
ot_relion_project.py

ot-tools script

Click here to access/download
Supplementary Material
ot_remap.py

ot-tools script

Click here to access/download

Supplementary Material

ot_rot-ps.py

Click here to access/download
Supplementary Material
ot_unskew.py

Dear Dr. Nogoy,

Thank you for editing our manuscript. We thank the reviewers for their positive and constructive comments. Below, we provide a point-by-point rebuttal, with the reviewers’ original comments in black text and our replies in blue indented text. We have also addressed your editorial comments.

Sincerely,
Lu Gan

Editorial: I see you also have scripts in GitHub - (https://github.com/anaphaze/ot-tools) and these need an OSI (open source initiative) license assigned to them. Please choose an appropriate one and add this to GitHub.

In the repository, we have included a MIT license, which is OSI approved. This license is also included in the zipped archive that we uploaded in reply to Reviewer 1.

I have also added some minor formatting comments to your manuscript - please see the attached version and update this.

All of the suggested changes have been incorporated.

In addition, please register any new software application in the SciCrunch.org database to receive a RRID (Research Resource Identification Initiative ID) number, and include this in your manuscript. This will facilitate tracking, reproducibility and re-use of your tool.

Thank you for this suggestion. We have registered our Github ot-tools repository as RSCR_017191 and have added this RRID to the revised manuscript lines 309-311:

"Python scripts to help facilitate 3-D packing analysis of subtomograms are available in the ot-tools GitHub repository (RRID: SCR_017191) [28]."

In addition to addressing the editorial comments and reviews, we have made a few minor additions to reflect feedback on our preprint from colleagues in the field.

1) Following an interesting twitter discussion, we have added new details on how to access the EMPIAR data to lines 320-329:

"This data can be downloaded either using the Aspera Connect client or with a web browser. We do not recommend download by web browser due to its slow speed and lack of fault tolerance. Users of Unix-like operating systems, e.g., Linux and macOS, may also do bulk downloads with the program "wget" by running, as an example, the following command from the terminal:

```
wget -b -m -nH --cut-dirs=6
'ftp://ftp.ebi.ac.uk/pub/databases/empiar/archive/10227/data/US1363_G1/*_tilt'
```

This command will retrieve all of the US1363_G1 tilt series to the directory from which the command was executed."

2) We have also added 3 new citations to alert readers to *in situ* cryo-ET papers that have explored in greater detail some of the structures presented in Figures 2 and 3:

Cytokinetic machinery: Swulius *et al*, PNAS 2018
Intranuclear proteasomes: Albert *et al*, PNAS 2017
ER-PM connection: Hoffmann *et al*, bioRxiv 2019

Reviewer #1: This is an excellent Data Note that uses electron cryotomography to generate 3D volumetric images that reveal organelle and macromolecular complex-level structure in the budding yeast S. cerevisiae. The dataset, composed of 1,000 cryo-ET raw datasets, is neatly organised into 5 distinct subsets that reflect the strain ID plus either the cell-cycle state or treatment. Details of pixel size are provided in the manuscript and the accompanying metadata (Google sheets), enabling measurements to be made on the volumetric images. From a reuse perspective, I was particularly pleased that the authors explored angular rotation, which is often overlooked, as there are web-based visualisation tools such as IIP3D that could allow researchers to cut arbitrary sections through these 3D data volumes. I see great reuse potential in this dataset, and I congratulate the authors for bringing attention to this in the manuscript.

Thank you for bringing to our attention the IIP3D volumetric visualization tool, which we have added to our citation list of browsing tools lin line 283-284.

From a data storage perspective, the EM images are all deposited in EMPIAR, which is the EBI resource for raw, 2D electron microscopy images. Importantly, a DOI has been ascribed to this dataset and so there is no need for the GigaScience DataBase to take a copy of the image files. The metadata are made available through Google sheets and these have allowed me to observe that these data are organised in an orderly manner that could facilitate re-use.

We thank the Reviewer for these positive comments. The sharing of surplus cellular cryo-ET data is a new phenomenon. We hope this work and the one from the Jensen lab (Ortega *et al.* 2019, Plos ONE) will encourage others in the field to do the same. We also hope this manuscript will stimulate feedback from the broader cell-biology community, which we can use to improve future data depositions and metadata presentation. As an example, Reviewer 2 has already suggested how anyone in the world is free to re-share our dataset as an ETDB database.

However, I do request that the authors additionally submit these metadata to the GigaScience DataBase as tabular data (comma-separated file format) to ensure long-term access.

We have now uploaded the metadata spreadsheets in .xlsx format to GigaScience. We will work with the GigaScience production staff if they require any modifications or alternative file formats. We have also uploaded a copy of the .xlsx file to EMPIAR-10227, as requested by Reviewer 2. In the revised text, each reference to the location of these metadata now also cite the copy at EMPIAR and GigaDB.

Furthermore, I suggest that a snapshot of the GutHub archive, which includes python scripts used in 3D analysis of the cryo-ET data (https://github.com/anaphaze/ot-tools), is also submitted to the GigaScience DataBase. Importantly, this GitHub archive has an OSI-approved MIT permissive free software license and therefore is open and available for reuse.

We have downloaded a copy of the GitHub archive and included it with this revision.

As a minor point, the authors make the following statement about future work:
"The current entry does not include any movie or electron-counted data. In the future, electron-counted raw data will be stored as LZW-compressed .tiff files." It would be helpful if the authors could clarify whether these data will be added to the existing EMPIAR dataset, or whether they intend on submitting these data to GigaScience.

This is a good question and one that we had hoped would be resolved by now. Last year, we asked EMPIAR if we could update EMPIAR-10227 with (much) more data in the future. We were asked to wait for a decision, but have not heard back yet. We reminded EMPIAR of our question just a couple weeks ago, but unfortunately, we still have not heard back. As a compromise, we have added this sentence to lines 130-132:

“If possible, we will add the newer data to the existing entry EMPIAR-10227. Otherwise, we will create new EMPIAR entries that each contains a link to this manuscript.”

Reviewer #2: This manuscript reports the deposition of over one thousand cryo-ET tilt-series of cryosections of budding yeast into a publicly-accessible database, EMPIAR. We agree with all the claims in the abstract, including that these tilt-series, cryotomograms, and metadata hold new information about numerous cellular structures and that these files will be useful in cryo-ET software development and training. We applaud the authors for sharing all this data with the community. The paper is well-written and clear, except for a few very minor issues (line 216). Metadata, including comments about each cryotomogram and notes about possible biological features present, are provided in public Google spreadsheets. Unfortunately many spreadsheet entries are cryptic, and there are unclear abbreviations (what do "N" and "need more dig" mean)?

> Thank you for pointing on the typo in line 216. The corrected sentence reads:
>
> "These evaluations were made from cryotomograms when possible."
>
> We have also corrected a few other typos and strange word choices throughout the text.
>
> The letter "N" is an abbreviation for nucleus in the "Remarks after reconstruction" and "Diagnostic remarks" columns. All of the abbreviations appear when the mouse is hovered over the google sheet notes, but we did not state this in the original manuscript. We have now pointed these notes in the revised manuscript, lines 157-160:
>
> "During the initial annotation, we used abbreviations to denote organelles and other cellular features. These abbreviations are defined in the spreadsheet "notes", which can be displayed by hovering the mouse cursor over the spreadsheet title cell."
>
> Phrases like "need more dig" (now removed) are either typos or personal abbreviations of the microscopists who did the initial annotation. During the preparation of this manuscript, we have spent considerable time making the annotations consistent and removing obvious typos. We are sure there are other small problems we have missed and we hope that readers will use the feedback form to bring these mistakes to our attention. We summarize these ideas in revised lines 143-145:
>
> "The Google Sheets are "live" documents and will be updated as new datasets are deposited and as errata are brought to our attention and then corrected."

We also wonder why the authors put the metadata spreadsheets on Google rather than simply adding them to the EMPIAR folders where we think they would be more easily found.

> We prefer Google Sheets because it is much easier and faster for us to curate the metadata on this resource. Nevertheless the Reviewers' question led us to the realization that Google services are blocked in some countries. To increase accessibility, we have uploaded the Excel form of the metadata to EMPIAR. We believe that in the future, we will be able to update the EMPIAR copy of the metadata, albeit with less frequency.

We agree with the authors that archiving cryo-ET datasets on resources such as EMPIAR is wonderful, but we would like to point out some advantages and disadvantages of this particular route. We've thought about this quite a bit, since we recently designed and built our own, different strategy for the same purpose (in our case we shared over ten thousand tilt-series and

cryotomograms of bacterial cells, see Ortega, D.R. et al. 2019. ETDB-Caltech: A blockchain-based distributed public database for electron tomography. PLOS ONE, 14, e0215531). Our image files are stored both on our servers and possibly elsewhere on a public peer-to-peer distributed file system, and our metadata is published in the FLO blockchain. Together they form a resource we named the Electron Tomography Database (ETDB). Comparing the two approaches, we believe the main advantage of the EMPIAR and Google spreadsheets mechanism used by Gan et al. is that both resources (EMPIAR and Google spreadsheets) are already familiar to researchers in the field. This familiarity will lower the access barrier. The main disadvantage is, however, that it will be clumsy for folks who want to search for and download certain diverse tilt-series, since they will have to search multiple sheets in the Google spreadsheets for what they want, then navigate the custom folder structure of these authors' special EMPIAR deposition to get them. The problem compounds if multiple groups who want to share cryotomograms upload them in similar fashion (to EMPIAR in special folder trees with spreadsheets of metadata organized in unique ways and stored in diverse places). Searches and retrievals would no longer be easily scriptable. We solved these problems, while retaining full flexibility in what metadata and data files are stored by each depositor, by using the FLO blockchain as a flexible, public, and permanent distributed ledger that serves as a universal index. The blockchain ledger system is ownerless, permissionless, and independent of grant renewals to a host institution. Additional analyses (like automatic segmentations of the tomograms by others) can be added and linked to the original data by anyone at anytime. As far as we understand, with EMPIAR and Google spreadsheets this could only be done if segmentors added their own additional google spreadsheets and published additional EMPIAR entries, again increasing the complexity of searching and finding all related material to a particular tilt-series.

> We agree with every point raised here about the advantages and disadvantages of Google Sheets/EMPIAR versus ETDB. We hope that as more cryo-ET data becomes publicly available, either via EMPIAR+Google docs or ETDB, the cell biology community will use both resources and test the ideas put forth here. Perhaps an enterprising lab or individual will collect all the EMPIAR (and other publicly available) entries and create a super dataset using ETDB, as you have alluded to below.

What if ten different labs deposited cryotomography data, and then three separate software developers found ways to automatically segment cells in the cryotomograms, and each wanted to post their results linked to the original cryotomograms? Would they each have to ask all ten depositing labs permission to add their segmentation metadata to each lab's spreadsheet, or would they add their own spreadsheet referencing up to ten original spreadsheets found in potentially different places? Would they have to agree on the format of the new columns in the spreadsheet? How would the segmentations be linked to individual tomograms? ETDB's permissionless and ownerless distributed ledger (the FLO blockchain) is like a single extensible spreadsheet with metadata and permanent links to all the individual cryo-ET datasets contributed by any lab, that anyone can read and search in its entirety. Anyone can add new information to it without group debate, consensus or permission.

> This is a good example of how the ownerless nature of ETDB's metadata system allows for more flexibility in team-based annotations to a public resource.

All that being said, we view it as a great thing that different approaches are now being tried, and we note that it would be easy enough for anyone to add all of our data to EMPIAR, or add all of Gan et al.'s data to the ETDB, so time and experience will ultimately reveal which methods are best. In summary, this excellent dataset presented by the Gan Lab is a unique and valuable

resource for the study of yeast ultrastructure, further development of cryo-ET software, and training.

We completely agree that these datasets can now be duplicated and therefore coexist in both the EMPIAR and ETDB systems. Our university is quite restrictive about exposing servers to the internet, so it was important for us to make our data available by a method we had already tested. Now that our data is on EMPIAR, a lab from a less-restrictive university could indeed convert EMPIAR-10227 into an ETDB database. In new lines 312 -314, we encourage this action:

“We note that anyone can add our data to an ETDB database [14] and thereby enable the numerous benefits of ownerless-ledger metadata and decentralized storage.”
