# Supplementary material for: A collection of yeast cellular electron cryotomography data
Source: Gigascience. 2019 Jun 27;8(6):giz077. doi: 10.1093/gigascience/giz077 (PMC6596884; doi:10.1093/gigascience/giz077)
Supplement: giz077_Response_to_Reviewer_Comments_Original_Submission [file giz077_response_to_reviewer_comments_original_submission.pdf]

Author's Response To Reviewer Comments

Close

Dear Dr. Nogoy,

Our rebuttal was formatted such that our comments were in blue indented text. However, this formatting could not be transferred to this "Respond to Reviewers" page. I hope that the Reviewers will be able to see our separate rebuttal PDF file, which is correctly formatted.

Thanks.
Lu

Dear Dr. Nogoy,

Thank you for editing our manuscript. We thank the reviewers for their positive and constructive comments. Below, we provide a point-by-point rebuttal, with the reviewers' original comments in black text and our replies in blue indented text. We have also addressed your editorial comments.

Sincerely,
Lu Gan
Editorial: I see you also have scripts in GitHub - (https://github.com/anaphaze/ot-tools) and these need an OSI (open source initiative) license assigned to them. Please choose an appropriate one and add this to GitHub.

In the repository, we have included a MIT license, which is OSI approved. This license is also included in the zipped archive that we uploaded in reply to Reviewer 1.

I have also added some minor formatting comments to your manuscript - please see the attached version and update this.

All of the suggested changes have been incorporated.

In addition, please register any new software application in the SciCrunch.org database to receive a RRID (Research Resource Identification Initiative ID) number, and include this in your manuscript. This will facilitate tracking, reproducibility and re-use of your tool.

Thank you for this suggestion. We have registered our Github ot-tools repository as RSCR_017191 and have added this RRID to the revised manuscript lines 309-311:

"Python scripts to help facilitate 3-D packing analysis of subtomograms are available in the ot-tools GitHub repository (RRID: SCR_017191) [28]."

In addition to addressing the editorial comments and reviews, we have made a few minor additions to reflect feedback on our preprint from colleagues in the field.

1) Following an interesting twitter discussion, we have added new details on how to access the EMPIAR data to lines 320-329:

"This data can be downloaded either using the Aspera Connect client or with a web browser. We do not recommend download by web browser due to its slow speed and lack of fault tolerance. Users of Unix-like operating systems, e.g., Linux and macOS, may also do bulk downloads with the program "wget" by running, as an example, the following command from the terminal:

```
wget -b -m -nH --cut-dirs=6
'ftp://ftp.ebi.ac.uk/pub/databases/empiar/archive/10227/data/US1363_G1/*_tilt'
```

This command will retrieve all of the US1363_G1 tilt series to the directory from which the command was executed."

2) We have also added 3 new citations to alert readers to in situ cryo-ET papers that have explored in greater detail some of the structures presented in Figures 2 and 3:

Cytokinetic machinery: Swulius et al, PNAS 2018
Intranuclear proteasomes: Albert et al, PNAS 2017
ER-PM connection: Hoffmann et al, bioRxiv 2019

Reviewer #1: This is an excellent Data Note that uses electron cryotomography to generate 3D volumetric images that reveal organelle and macromolecular complex-level structure in the budding yeast S. cerevisiae. The dataset, composed of 1,000 cryo-ET raw datasets, is neatly organised into 5 distinct subsets that reflect the strain ID plus either the cell-cycle state or treatment. Details of pixel size are provided in the manuscript and the accompanying metadata (Google sheets), enabling measurements to be made on the volumetric images. From a reuse perspective, I was particularly pleased that the authors explored angular rotation, which is often overlooked, as there are web-based visualisation tools such as IIP3D that could allow researchers to cut arbitrary sections through these 3D data volumes. I see great reuse potential in this dataset, and I congratulate the authors for bringing attention to this in the manuscript.

Thank you for bringing to our attention the IIP3D volumetric visualization tool, which we have added to our citation list of browsing tools lin line 283-284.

From a data storage perspective, the EM images are all deposited in EMPIAR, which is the EBI resource for raw, 2D electron microscopy images. Importantly, a DOI has been ascribed to this dataset and so there is no need for the GigaScience DataBase to take a copy of the image files. The metadata are made available through Google sheets and these have allowed me to observe that these data are organised in an orderly manner that could facilitate re-use.

We thank the Reviewer for these positive comments. The sharing of surplus cellular cryo-ET data is a new phenomenon. We hope this work and the one from the Jensen lab (Ortega et al. 2019, Plos ONE) will encourage others in the field to do the same. We also hope this manuscript will stimulate feedback from the broader cell-biology community, which we can use to improve future data depositions and metadata presentation. As an example, Reviewer 2 has already suggested how anyone in the world is free to re-share our dataset as an ETDB database.

However, I do request that the authors additionally submit these metadata to the GigaScience DataBase as tabular data (comma-separated file format) to ensure long-term access.

We have now uploaded the metadata spreadsheets in .xlsx format to GigaScience. We will work with the GigaScience production staff if they require any modifications or alternative file formats. We have also uploaded a copy of the .xlsx file to EMPIAR-10227, as requested by Reviewer 2. In the revised text, each reference to the location of these metadata now also cite the copy at EMPIAR and GigaDB.

Furthermore, I suggest that a snapshot of the GutHub archive, which includes python scripts used in 3D analysis of the cryo-ET data (https://github.com/anaphaze/ot-tools), is also submitted to the GigaScience DataBase. Importantly, this GitHub archive has an OSI-approved MIT permissive free software license and therefore is open and available for reuse.

We have downloaded a copy of the GitHub archive and included it with this revision.

As a minor point, the authors make the following statement about future work:
"The current entry does not include any movie or electron-counted data. In the future, electron-counted raw data will be stored as LZW-compressed .tiff files." It would be helpful if the authors could clarify whether these data will be added to the existing EMPIAR dataset, or whether they intend on submitting these data to GigaScience.

This is a good question and one that we had hoped would be resolved by now. Last year, we asked EMPIAR if we could update EMPIAR-10227 with (much) more data in the future. We were asked to wait for a decision, but have not heard back yet. We reminded EMPIAR of our question just a couple weeks

ago, but unfortunately, we still have not heard back. As a compromise, we have added this sentence to lines 130-132:

“If possible, we will add the newer data to the existing entry EMPIAR-10227. Otherwise, we will create new EMPIAR entries that each contains a link to this manuscript.”
Reviewer #2: This manuscript reports the deposition of over one thousand cryo-ET tilt-series of cryosections of budding yeast into a publicly-accessible database, EMPIAR. We agree with all the claims in the abstract, including that these tilt-series, cryotomograms, and metadata hold new information about numerous cellular structures and that these files will be useful in cryo-ET software development and training. We applaud the authors for sharing all this data with the community. The paper is well-written and clear, except for a few very minor issues (line 216). Metadata, including comments about each cryotomogram and notes about possible biological features present, are provided in public Google spreadsheets. Unfortunately many spreadsheet entries are cryptic, and there are unclear abbreviations (what do "N" and "need more dig" mean)?

Thank you for pointing on the typo in line 216. The corrected sentence reads:

“These evaluations were made from cryotomograms when possible.”

We have also corrected a few other typos and strange word choices throughout the text.

The letter “N” is an abbreviation for nucleus in the “Remarks after reconstruction” and “Diagnostic remarks” columns. All of the abbreviations appear when the mouse is hovered over the google sheet notes, but we did not state this in the original manuscript. We have now pointed these notes in the revised manuscript, lines 157-160:

“During the initial annotation, we used abbreviations to denote organelles and other cellular features. These abbreviations are defined in the spreadsheet “notes”, which can be displayed by hovering the mouse cursor over the spreadsheet title cell.”

Phrases like “need more dig” (now removed) are either typos or personal abbreviations of the microscopists who did the initial annotation. During the preparation of this manuscript, we have spent considerable time making the annotations consistent and removing obvious typos. We are sure there are other small problems we have missed and we hope that readers will use the feedback form to bring these mistakes to our attention. We summarize these ideas in revised lines 143-145:

“The Google Sheets are “live” documents and will be updated as new datasets are deposited and as errata are brought to our attention and then corrected.”

We also wonder why the authors put the metadata spreadsheets on Google rather than simply adding them to the EMPIAR folders where we think they would be more easily found.

We prefer Google Sheets because it is much easier and faster for us to curate the metadata on this resource. Nevertheless the Reviewers’ question led us to the realization that Google services are blocked in some countries. To increase accessibility, we have uploaded the Excel form of the metadata to EMPIAR. We believe that in the future, we will be able to update the EMPIAR copy of the metadata, albeit with less frequency.

We agree with the authors that archiving cryo-ET datasets on resources such as EMPIAR is wonderful, but we would like to point out some advantages and disadvantages of this particular route. We've thought about this quite a bit, since we recently designed and built our own, different strategy for the same purpose (in our case we shared over ten thousand tilt-series and cryotomograms of bacterial cells, see Ortega, D.R. et al. 2019. ETDB-Caltech: A blockchain-based distributed public database for electron tomography. PLOS ONE, 14, e0215531). Our image files are stored both on our servers and possibly elsewhere on a public peer-to-peer distributed file system, and our metadata is published in the FLO blockchain. Together they form a resource we named the Electron Tomography Database (ETDB). Comparing the two approaches, we believe the main advantage of the EMPIAR and Google spreadsheets mechanism used by Gan et al. is that both resources (EMPIAR and Google spreadsheets) are already familiar to researchers in the field. This familiarity will lower the access barrier. The main disadvantage is, however, that it will be clumsy for folks who want to search for and download certain diverse tilt-series, since they will have to search multiple sheets in the Google spreadsheets for what they want,

then navigate the custom folder structure of these authors' special EMPIAR deposition to get them. The problem compounds if multiple groups who want to share cryotomograms upload them in similar fashion (to EMPIAR in special folder trees with spreadsheets of metadata organized in unique ways and stored in diverse places). Searches and retrievals would no longer be easily scriptable. We solved these problems, while retaining full flexibility in what metadata and data files are stored by each depositor, by using the FLO blockchain as a flexible, public, and permanent distributed ledger that serves as a universal index. The blockchain ledger system is ownerless, permissionless, and independent of grant renewals to a host institution. Additional analyses (like automatic segmentations of the tomograms by others) can be added and linked to the original data by anyone at anytime. As far as we understand, with EMPIAR and Google spreadsheets this could only be done if segmentors added their own additional google spreadsheets and published additional EMPIAR entries, again increasing the complexity of searching and finding all related material to a particular tilt-series.

We agree with every point raised here about the advantages and disadvantages of Google Sheets/EMPIAR versus ETDB. We hope that as more cryo-ET data becomes publicly available, either via EMPIAR+Google docs or ETDB, the cell biology community will use both resources and test the ideas put forth here. Perhaps an enterprising lab or individual will collect all the EMPIAR (and other publicly available) entries and create a super dataset using ETDB, as you have alluded to below.

What if ten different labs deposited cryotomography data, and then three separate software developers found ways to automatically segment cells in the cryotomograms, and each wanted to post their results linked to the original cryotomograms? Would they each have to ask all ten depositing labs permission to add their segmentation metadata to each lab's spreadsheet, or would they add their own spreadsheet referencing up to ten original spreadsheets found in potentially different places? Would they have to agree on the format of the new columns in the spreadsheet? How would the segmentations be linked to individual tomograms? ETDB's permissionless and ownerless distributed ledger (the FLO blockchain) is like a single extensible spreadsheet with metadata and permanent links to all the individual cryo-ET datasets contributed by any lab, that anyone can read and search in its entirety. Anyone can add new information to it without group debate, consensus or permission.

This is a good example of how the ownerless nature of ETDB's metadata system allows for more flexibility in team-based annotations to a public resource.

All that being said, we view it as a great thing that different approaches are now being tried, and we note that it would be easy enough for anyone to add all of our data to EMPIAR, or add all of Gan et al.'s data to the ETDB, so time and experience will ultimately reveal which methods are best. In summary, this excellent dataset presented by the Gan Lab is a unique and valuable resource for the study of yeast ultrastructure, further development of cryo-ET software, and training.

We completely agree that these datasets can now be duplicated and therefore coexist in both the EMPIAR and ETDB systems. Our university is quite restrictive about exposing servers to the internet, so it was important for us to make our data available by a method we had already tested. Now that our data is on EMPIAR, a lab from a less-restrictive university could indeed convert EMPIAR-10227 into an ETDB database. In new lines 312 -314, we encourage this action:

"We note that anyone can add our data to an ETDB database [14] and thereby enable the numerous benefits of ownerless-ledger metadata and decentralized storage."

Close
