# Supplementary material for: A collection of yeast cellular electron cryotomography data
Source: Gigascience. 2019 Jun 27;8(6):giz077. doi: 10.1093/gigascience/giz077 (PMC6596884; doi:10.1093/gigascience/giz077)
Supplement: giz077_Reviewer_1_Report_Original_Submission — Chris Armit -- 4/5/2019 Reviewed [file giz077_reviewer_1_report_original_submission.pdf]

Reviewer Report

Title: A collection of yeast cellular electron cryotomography data

Version: Original Submission **Date:** 4/5/2019

Reviewer name: Chris Armit

Reviewer Comments to Author:

This is an excellent Data Note that uses electron cryotomography to generate 3D volumetric images that reveal organelle and macromolecular complex-level structure in the budding yeast S. cerevisiae. The dataset, composed of 1,000 cryo-ET raw datasets, is neatly organised into 5 distinct subsets that reflect the strain ID plus either the cell-cycle state or treatment. Details of pixel size are provided in the manuscript and the accompanying metadata (Google sheets), enabling measurements to be made on the volumetric images. From a reuse perspective, I was particularly pleased that the authors explored angular rotation, which is often overlooked, as there are web-based visualisation tools such as IIP3D that could allow researchers to cut arbitrary sections through these 3D data volumes. I see great reuse potential in this dataset, and I congratulate the authors for bringing attention to this in the manuscript. From a data storage perspective, the EM images are all deposited in EMPIAR, which is the EBI resource for raw, 2D electron microscopy images. Importantly, a DOI has been ascribed to this dataset and so there is no need for the GigaScience DataBase to take a copy of the image files. The metadata are made available through Google sheets and these have allowed me to observe that these data are organised in an orderly manner that could facilitate re-use. However, I do request that the authors additionally submit these metadata to the GigaScience DataBase as tabular data (comma-separated file format) to ensure long-term access. Furthermore, I suggest that a snapshot of the GutHub archive, which includes python scripts used in 3D analysis of the cryo-ET data (https://github.com/anaphaze/ot-tools), is also submitted to the GigaScience DataBase. Importantly, this GitHub archive has an OSI-approved MIT permissive free software license and therefore is open and available for reuse.
As a minor point, the authors make the following statement about future work:
"The current entry does not include any movie or electron-counted data. In the future, electron-counted raw data will be stored as LZW-compressed .tiff files."
It would be helpful if the authors could clarify whether these data will be added to the existing EMPIAR dataset, or whether they intend on submitting these data to GigaScience.

Level of Interest

Please indicate how interesting you found the manuscript: Choose an item.

Quality of Written English

Please indicate the quality of language in the manuscript: Choose an item.

Declaration of Competing Interests

Please complete a declaration of competing interests, considering the following questions:

- Have you in the past five years received reimbursements, fees, funding, or salary from an organisation that may in any way gain or lose financially from the publication of this manuscript, either now or in the future?
- Do you hold any stocks or shares in an organisation that may in any way gain or lose financially from the publication of this manuscript, either now or in the future?
- Do you hold or are you currently applying for any patents relating to the content of the manuscript?
- Have you received reimbursements, fees, funding, or salary from an organization that holds or has applied for patents relating to the content of the manuscript?
- Do you have any other financial competing interests?
- Do you have any non-financial competing interests in relation to this paper?

If you can answer no to all of the above, write 'I declare that I have no competing interests' below. If your reply is yes to any, please give details below.

I declare that I have no competing interests.

I agree to the open peer review policy of the journal. I understand that my name will be included on my report to the authors and, if the manuscript is accepted for publication, my named report including any attachments I upload will be posted on the website along with the authors' responses. I agree for my report to be made available under an Open Access Creative Commons CC-BY license (http://creativecommons.org/licenses/by/4.0/). I understand that any comments which I do not wish to be included in my named report can be included as confidential comments to the editors, which will not be published.

I agree to the open peer review policy of the journal

To further support our reviewers, we have joined with Publons, where you can gain additional credit to further highlight your hard work (see: https://publons.com/journal/530/gigascience). On publication of this paper, your review will be automatically added to Publons, you can then choose whether or not to claim your Publons credit. I understand this statement.

Yes Choose an item.
