# Supplementary material for: A collection of yeast cellular electron cryotomography data
Source: Gigascience. 2019 Jun 27;8(6):giz077. doi: 10.1093/gigascience/giz077 (PMC6596884; doi:10.1093/gigascience/giz077)
Supplement: giz077_Reviewer_2_Report_Original_Submission — Grant Jensen -- 4/19/2019 Reviewed [file giz077_reviewer_2_report_original_submission.pdf]

Reviewer Report

Title: A collection of yeast cellular electron cryotomography data

Version: Original Submission **Date:** 4/19/2019

Reviewer name: Grant Jensen

Reviewer Comments to Author:

This manuscript reports the deposition of over one thousand cryo-ET tilt-series of cryosections of budding yeast into a publicly-accessible database, EMPIAR. We agree with all the claims in the abstract, including that these tilt-series, cryotomograms, and metadata hold new information about numerous cellular structures and that these files will be useful in cryo-ET software development and training. We applaud the authors for sharing all this data with the community. The paper is well-written and clear, except for a few very minor issues (line 216). Metadata, including comments about each cryotomogram and notes about possible biological features present, are provided in public Google spreadsheets. Unfortunately many spreadsheet entries are cryptic, and there are unclear abbreviations (what do "N" and "need more dig" mean)? We also wonder why the authors put the metadata spreadsheets on Google rather than simply adding them to the EMPIAR folders where we think they would be more easily found.

We agree with the authors that archiving cryo-ET datasets on resources such as EMPIAR is wonderful, but we would like to point out some advantages and disadvantages of this particular route. We've thought about this quite a bit, since we recently designed and built our own, different strategy for the same purpose (in our case we shared over ten thousand tilt-series and cryotomograms of bacterial cells, see Ortega, D.R. et al. 2019. ETDB-Caltech: A blockchain-based distributed public database for electron tomography. PLOS ONE, 14, e0215531). Our image files are stored both on our servers and possibly elsewhere on a public peer-to-peer distributed file system, and our metadata is published in the FLO blockchain. Together they form a resource we named the Electron Tomography Database (ETDB). Comparing the two approaches, we believe the main advantage of the EMPIAR and Google spreadsheets mechanism used by Gan et al. is that both resources (EMPIAR and Google spreadsheets) are already familiar to researchers in the field. This familiarity will lower the access barrier. The main disadvantage is, however, that it will be clumsy for folks who want to search for and download certain diverse tilt-series, since they will have to search multiple sheets in the Google spreadsheets for what they want, then navigate the custom folder structure of these authors' special EMPIAR deposition to get them. The problem compounds if multiple groups who want to share cryotomograms upload them in similar fashion (to EMPIAR in special folder trees with spreadsheets of metadata organized in unique ways and stored in diverse places). Searches and retrievals would no longer be easily scriptable. We solved these problems, while retaining full flexibility in what metadata and data files are stored by each depositor, by using the FLO blockchain as a flexible, public, and permanent distributed ledger that serves as a universal index. The blockchain ledger system is ownerless, permissionless, and independent of grant renewals to a host institution. Additional analyses (like automatic segmentations of the tomograms by others) can be added and linked to the original data by anyone at anytime. As far as we understand,

with EMPIAR and Google spreadsheets this could only be done if segmentors added their own additional google spreadsheets and published additional EMPIAR entries, again increasing the complexity of searching and finding all related material to a particular tilt-series.

What if ten different labs deposited cryotomography data, and then three separate software developers found ways to automatically segment cells in the cryotomograms, and each wanted to post their results linked to the original cryotomograms? Would they each have to ask all ten depositing labs permission to add their segmentation metadata to each lab's spreadsheet, or would they add their own spreadsheet referencing up to ten original spreadsheets found in potentially different places? Would they have to agree on the format of the new columns in the spreadsheet? How would the segmentations be linked to individual tomograms? ETDB's permissionless and ownerless distributed ledger (the FLO blockchain) is like a single extensible spreadsheet with metadata and permanent links to all the individual cryo-ET datasets contributed by any lab, that anyone can read and search in its entirety. Anyone can add new information to it without group debate, consensus or permission.

All that being said, we view it as a great thing that different approaches are now being tried, and we note that it would be easy enough for anyone to add all of our data to EMPIAR, or add all of Gan et al.'s data to the ETDB, so time and experience will ultimately reveal which methods are best. In summary, this excellent dataset presented by the Gan Lab is a unique and valuable resource for the study of yeast ultrastructure, further development of cryo-ET software, and training.

Grant Jensen
Davi Ortega

Level of Interest

Please indicate how interesting you found the manuscript: Choose an item.

Quality of Written English

Please indicate the quality of language in the manuscript: Choose an item.

Declaration of Competing Interests

Please complete a declaration of competing interests, considering the following questions:

- Have you in the past five years received reimbursements, fees, funding, or salary from an organisation that may in any way gain or lose financially from the publication of this manuscript, either now or in the future?
- Do you hold any stocks or shares in an organisation that may in any way gain or lose financially from the publication of this manuscript, either now or in the future?
- Do you hold or are you currently applying for any patents relating to the content of the manuscript?
- Have you received reimbursements, fees, funding, or salary from an organization that holds or has applied for patents relating to the content of the manuscript?

- Do you have any other financial competing interests?
- Do you have any non-financial competing interests in relation to this paper?

If you can answer no to all of the above, write 'I declare that I have no competing interests' below. If your reply is yes to any, please give details below.

I declare that I have no competing interests

I agree to the open peer review policy of the journal. I understand that my name will be included on my report to the authors and, if the manuscript is accepted for publication, my named report including any attachments I upload will be posted on the website along with the authors' responses. I agree for my report to be made available under an Open Access Creative Commons CC-BY license (http://creativecommons.org/licenses/by/4.0/). I understand that any comments which I do not wish to be included in my named report can be included as confidential comments to the editors, which will not be published.

I agree to the open peer review policy of the journal

To further support our reviewers, we have joined with Publons, where you can gain additional credit to further highlight your hard work (see: https://publons.com/journal/530/gigascience). On publication of this paper, your review will be automatically added to Publons, you can then choose whether or not to claim your Publons credit. I understand this statement.

Yes Choose an item.
